# Species review of the genus *Boreophilia* Benick from North America (Coleoptera, Staphylinidae, Aleocharinae, Athetini): Systematics, habitat, and distribution

**DOI:** 10.3897/zookeys.848.34846

**Published:** 2019-05-20

**Authors:** Jan Klimaszewski, Derek S. Sikes, Adam Brunke, Caroline Bourdon

**Affiliations:** 1 Natural Resources Canada, Canadian Forest Service, Laurentian Forestry Centre, 1055 du PEPS, PO Box 10380, Stn. Sainte-Foy, Québec, QC, G1V 4C7, Canada Natural Resources Canada Québec Canada; 2 University of Alaska Museum, 1962 Yukon Drive, Fairbanks, Alaska 99775-6960, USA University of Alaska Fairbanks United States of America; 3 Canadian National Collection of Insects, Agriculture and Agri-Food Canada, Ottawa, ON, K1A 0C6, Canada Agriculture and Agri-Food Canada Ottawa Canada

**Keywords:** Identification, nomenclature, North America, northern species, rove beetles, taxonomy

## Abstract

Fourteen species of the genus *Boreophilia* Benick are now recognized in North America. *Boreophiliainsecuta* (Eppelsheim), reported by Lohse (1990) from North America, is a misidentification of a new species, which is described here as *B.neoinsecuta* Klimaszewski, **sp. n.**, and the true *B.insecuta* (Epp.) does not occur in North America. An additional new species is found in Alaska, and described as *B.beringi* Klimaszewski & Brunke, **sp. n.** The following three species are synonymized (second name being valid): *Boreophiliaherschelensis* Klimaszewski & Godin, 2012, with *Boreophiliavega* (Fenyes, 1920); *Boreophiliamanitobensis* Lohse, 1990, with *B.caseyi* Lohse, 1990; and *B.angusticornis* (Bernahuer, 1907) with *B.subplana* (J Sahlberg, 1880), based on study of genital structures and external morphology. *Athetagelida* J Sahlberg, 1887, and *Athetamunsteri* Bernhauer, 1902, considered as *Boreophilia* in recent publications, are transferred to the genus Atheta Thomson, subgenus Dimetrota. *Boreostibapiligera* (J Sahlberg) is transferred to *Boreophilia* based on morphology and the results of our phylogenetic analysis. *Boreophilianearctica* is recorded from Alberta and *B.nomensis* is recorded from British Columbia for the first time. Each valid species is illustrated by color image of habitus, and black and white images of genitalia and tergite and sternite VIII. A new key to all Nearctic species of the genus is provided. DNA barcode data were available for nine of the 14 species, which we downloaded, analyzed, and used as additional evidence for the taxonomic conclusions reached herein.

## Introduction

*Boreophilia* G Benick, 1973, is a small athetine genus, comprising Nordic species distributed exclusively in the Palaearctic and Nearctic regions. There are 17 species recorded in the Palaearctic (Smetana 2004), and 14 in the Nearctic regions. Of these, six species have a circumpolar Holarctic distribution, but two species included by Smetana (2004) in this genus, *B.gelida* (J Sahlberg) and *B.munsteri* (Bernhauer), are here transferred back to the genus *Atheta* where they were originally described, on the grounds of morphology of genital characters, including sexual modification of male tergite VIII, which is unmodified in *Boreophilia*. Consequently, there are 15 Palaearctic and 14 Nearctic species, of which six are Holarctic. It is interesting that none of the species with elytra shorter than the pronotum (and presumably brachypterous), were shown to be Holarctic. Limited dispersal in these groups has likely led to allopatric speciation between Nearctic and Palaearctic populations (e.g., *B.piligera* and *B.beringi* sp. n.). The Holarctic species constitute ca. 24% of the total fauna of the genus, which is likely the highest percentage of Holarctic species among Nearctic aleocharine genera. This genus, like *Gnypeta* CG Thomson, another northern Holarctic genus, is a good target for monitoring climate warming and its effects on distribution and survival of Nordic species. We here update our knowledge on all recorded Nearctic species and synonymize four species from previous records (Lohse et al. 1990, Klimaszewski et al. 2012). Two Nearctic species are described as new. All Nearctic species are classified to species groups, which presumably reflect their close relationships.

In the past, there was confusion regarding some Nearctic species of *Boreophilia* because species of this genus have similar structures of the median lobe of the aedeagus and of the spermatheca, insufficient material was available for study, and a general poor knowledge of Palaearctic species in the Nearctic region. We have corrected these as much as the available material permitted and have provided better diagnoses for Nearctic species. We have also studied European material to compare with Nearctic specimens of selected Holarctic species. This resulted in additional synonymy and clarification as to the known distribution of many species in North America. Brundin’s (1954) paper was very helpful to our study. This work was clearly ahead of its time, with perfectly accurate illustrations of the median lobe of the aedeagus and spermatheca of several European species, providing the best diagnostic characters at the species level. We hope to encourage other scientists to use species of this genus in monitoring the effects of climate change on species in Nordic environments.

## Material and methods

Almost all specimens used in this study were dissected, and their genital structures examined. The genital structures were dehydrated in absolute ethanol and mounted in Canada balsam on celluloid microslides, and pinned with the specimens from which they originated. The photographs of the entire body and the genital structures were taken using an image processing system (Nikon SMZ 1500 stereoscopic microscope; Nikon Digital Camera DXM 1200F; and Adobe Photoshop software).

Terminology mainly follows that used by Lohse et al. (1990) and Klimaszewski et al. 2018. The ventral part of the median lobe of the aedeagus is considered to be the part of the bulbus containing the foramen mediale, the entrance of the ductus ejaculatorius, and the adjacent venter (ventral part of the tubus of the median lobe) of the tubus; the opposite side is referred to as the dorsal part.

Depository abbreviations:

**CBG** Centre for Biodiversity Genomics, Guelph, Ontario, Canada

**CNC** Canadian National Collection of Insects, Arachnids and Nematodes, Ottawa, Ontario, Canada

**DEI** Deutsches Entomologisches Institut, Eberswalde, Germany

**LFC** Natural Resources Canada, Canadian Forest Service, Laurentian Forestry Centre, Insectarium R Martineau, Quebec City, Quebec, Canada

**NFRC** Natural Resources Canada, Canadian Forest Service, Northern Forestry Centre Arthropod Collection, Edmonton, Alberta, Canada

**MHNG** Muséum d’histoire naturelle, Genéve, Switzerland

**NHMD** University of Copenhagen, Copenhagen, Denmark

**RWC** Reginald Webster private collection, 24 Millstream Drive, Charters Settlement, New Brunswick, Canada

**UAM** University of Alaska Museum, Fairbanks, Alaska, United States of America

**USNM** United States National Museum (Smithsonian Institution), Washington D.C., United States of America

**ZMH** Zoological Museum, Helsinki, Finland

**ZMUO** Zoology Museum, University of Oulu, Oulu, Finland

DNA barcode data were downloaded from the BOLD website (http://www.boldsystems.org) after applying filters to exclude those flagged as misidentifications, those with sequence lengths under 100 bp, those with stop codons, and those flagged as contaminated. This resulted in sequence data for nine of the 14 species included herein. The amino acid based HMM BOLD aligner was used to align the data prior to download. Two sequences each of *Athetacinnamoptera* and *Athetamunsteri* were used as outgroups. The latter species was also included to test its generic placement. This resulted in a dataset of 33 sequences. Of 654 base pairs in the alignment, 455 are constant, 19 are variable but parsimony uninformative, and 180 are parsimony-informative. Specimens of all included *Boreophilia* were identified to species via morphological study, or to genus for some females. These sequences came from a variety of projects (Table 1) and publications (Elven et al. 2010, Pentinsaari et al. 2014, Sikes et al. 2017). The NEXUS file with the alignment and resulting tree is available for download from https://doi.org/10.6084/m9.figshare.7822496.

**Table 1.** DNA voucher data with Process ID codes from the Barcode of Life Database (BOLD), BOLD BIN numbers, sequence length with number of ‘n’s indicated, GenBank accession codes, and locality data. See http://www.boldsystems.org for additional data associated with each.

Identification	Process ID	BIN	Seq. Length	GenBank	Country/Ocean, State/Province, Region, Sector, Exact Site, Lat, Lon
*Athetacinnamoptera*	COLFC200-12	BOLD:ABW4507	658[0n]	KJ964314	Finland, Lapland, Lapponia kemensis pars orientalis, Sodankylae, Vuotso, 68.1117, 27.1862
	COLFC205-12	BOLD:ABW4507	614[0n]	KJ961954	Finland, Lapland, Lapponia kemensis pars orientalis, Sodankylae, Vuotso, 68.1117, 27.1862
*Athetamunsteri*	COLFA072-10	BOLD:AAJ9581	658[0n]	HM909090	Finland, Lapland, Lapponia enontekiensis, Enontekioe, 69.096, 21.138
	LEFIJ2464-14	BOLD:AAJ9581	658[0n]		Finland, Lapponia inarensis, Utsjoki, Skalluvaara, 69.802, 27.102
*Boreophilia* sp.	SAPIT188-08	BOLD:AAH0226	577[0n]		Canada, Manitoba, Churchill, 23 km E Churchill, Malcolm Ramsay Lake, road, Shrub community dominated by *Betulaglandulosa*, 58.73, -93.8
	UAMIC2716-15	BOLD:ACU9385	407[0n]	KU874453	United States, Alaska, Nogahabara Dunes [Koyukuk NWR], 65.658, -157.476
	TWCOL345-09	BOLD:AAG4312	658[0n]	HM432945	Canada, Manitoba, Churchill, 4 km SE Churchill, Dene Village, 58.734, -94.112
*Boreophiliaeremita*	COLFA420-12	BOLD:ABW4331	658[0n]	KJ963286	Finland, Northern Ostrobothnia, Ostrobottnia borealis pars australis, Kiiminki, 65.116, 25.829
	COLFB787-12	BOLD:ABW4331	658[0n]	KJ964811	Finland, Lapland, Lapponia inarensis, Utsjoki, Gaskabeaicohkka, 70.0088, 27.5069
	COLFE1022-13	BOLD:ABW4331	658[0n]	KJ965816	Finland, Ostrobottnia borealis pars borealis, Tornio, Alkunkarinlahti, 65.7811, 24.2119
	COLFB791-12	BOLD:ABW4331	583[0n]	KJ966458	Finland, Lapland, Lapponia inarensis, Utsjoki, Gaskabeaicohkka, 70.0088, 27.5069
	COLFB788-12	BOLD:ABW4331	582[0n]	KJ966313	Finland, Lapland, Lapponia inarensis, Utsjoki, Gaskabeaicohkka, 70.0088, 27.5069
	COLFB785-12	BOLD:ABW4331	567[2n]	KJ965976	Finland, Lapland, Lapponia inarensis, Utsjoki, Gaskabeaicohkka, 70.0088, 27.5069
*Boreophiliafusca*	COLFG320-14	BOLD:AAG4311	658[0n]		Finland, Lapponia inarensis, Inari, Kaamanen, 69.089, 27.184
	TWCOL344-09	BOLD:AAG4311	561[0n]	HM432944	Canada, Manitoba, Churchill, 4 km SE Churchill, Dene Village, 58.734, -94.112
*Boreophiliavega*	LFCAB223-15		407[0n]		Canada, Yukon Territory, Hershel Island, 69.571, -138.902
*Boreophiliahyperborea*	GBCL15075-13	BOLD:AAG4302	1000[1n]	GQ980933	Russia (specimen ZMUN:10002634)
	HMCOC722-09	BOLD:AAG4302	658[0n]	KJ203366	Canada, Manitoba, Churchill, 12 km S Churchill, Goose Creek Marina, Open substrate, 58.663, -94.166
*Boreophiliaislandica*	LFCAB221-15	BOLD:AAH0226	407[0n]		Canada, Newfoundland and Labrador, Long Range Mountains, Portland Creek Hill,
*Boreophilianearctica*	UAMIC2729-15	BOLD:ACU9385	658[0n]	KU874454	United States, Alaska, Naknek, 58.74, -157.064
	UAMIC2724-15	BOLD:ACU9385	613[0n]	KU874455	United States, Alaska, Selawik NWR, 66.561, -158.998
	LEPNG801-15	BOLD:ACU9385	658[0n]		Canada, Alberta, Plateau Mountain, 50.226, -114.555
	LEPNG802-15	BOLD:ACU9385	658[0n]		Canada, Alberta, Plateau Mountain, 50.226, -114.555
	LEPNG800-15	BOLD:ACU9385	407[0n]		Canada, Alberta, Plateau Mountain, 50.226, -114.555
*Boreophilianomensis*	UAMIC2675-15	BOLD:ACU9384	658[0n]	KU874456	United States, Alaska, Thompson Pass, 61.137, -145.745
	SSKNA9232-15	BOLD:ACU9384	564[0n]	MG057964	Canada, British Columbia, Kinaskan Lake Provincial Park, Kinaskan Lake Trail, 57.532, -130.202
*Boreophilia* sp.	UAMIC2676-15	BOLD:ACU9385	407[0n]	KU874457	United States, Alaska, Galena, Yukon Riv., W of town, 64.742, -156.98
	COLFC286-12	BOLD:ABX3767	658[0n]	KJ965200	Finland, Lapland, Lapponia inarensis, Inari, Saariselkae, 68.4214, 27.4396
*Boreophiliasubplana*	COLFB810-12		407[0n]	KJ962674	Finland, Lapland, Lapponia inarensis, Utsjoki, Gaskabeaicohkka, 70.0069, 27.5357
	COLFB811-12		407[0n]	KJ963490	Finland, Lapland, Lapponia inarensis, Utsjoki, Gaskabeaicohkka, 70.0069, 27.5357
*Boreophilianeoinsecuta*	MOBIL8660-18		545[0n]		United States, Alaska, Anaktuvuk Pass, 68.1405, -151.741
	MOBIL8661-18		492[0n]		United States, Alaska, Anaktuvuk Pass, 68.1405, -151.741
*Boreophiliapiligera*	COLFG746-14	BOLD:ACO9332	658[0n]		Finland, Lapponia enontekiensis, Enontekioe, Kilpisjaervi, Saana, 69.039, 20.854

To obtain a robust estimate of the mtDNA gene tree using these DNA barcode data, PartitionFinder2 (Lanfear et al. 2016) was used via the CIPRES Science Gateway (Miller et al. 2010) to obtain the best partitioning and modeling scheme. We used the following parameters for the cfg file: alignment = infile.phy, branchlengths = linked, models = all, model selection = aicc, search = greedy, with each codon position indicated as a separate partition. Mesquite v3.6 (Maddison and Maddison 2018), was used to export the original Nexus file to Phylip format for PartitionFinder. The best scheme chosen by PartitionFinder retained each codon position as a partition with first codon positions modeled using the TrN+I+G model, second positions modeled using the F81+I model, and third positions modeled using the GTR+G model. All DNA distances reported herein are uncorrected, p-distances. Minimum, average, and maximum distances were calculated in Excel from a distance matrix generated by PAUP 4.0a (build 164) (Swofford 2002). This data file is available at https://doi.org/10.6084/m9.figshare.7822508.

Bayesian and maximum likelihood phylogenetic analyses were conducted via the CIPRES portal using MrBayes v3.2.6 without the BEAGLE option (Ronquist et al. 2012) and Garli 2.0 (Zwickl 2006). Because MrBayes doesn’t have the TrN model, for the first codon position we used the GTR model, which PartitionFinder selected for use with MrBayes. Two runs of four chains each were sampled for 8 million generations with samples taken every 1000 generations; the first 25% of the samples were discarded as burn-in, yielding 12,002 samples. The average standard deviation of the split frequencies was 0.003158 and the average Potential Scale Reduction Factor (Gelman and Rubin 1992a, b) was 1.000, thus indicating convergence had been reached. The sampling was considered adequate based on the average estimated sample sizes (ESS) of the parameters all being greater than 2000, as assessed by MrBayes. Also using the CIPRES portal, we ran 200 bootstrap replicates composed of four search replicates each using GARLI, with zero length branches collapsed. The resulting trees were imported into PAUP 4.0a (build 164) (Swofford 2002) to produce a 50% majority rule consensus tree, the node support values of which were transferred to the Bayesian consensus tree. An additional maximum likelihood analysis was conducted in IQTREE 1.6 (Nguyen et al. 2015) on an iMac (4 GHz i7, 16GB) to acquire alternative node support values, namely the ultrafast bootstrap of Hoang et al. (2017) and the SH-aLRT test of Guindon et al. (2010). The analysis was performed using the same partitioning scheme as used for GARLI and with the -spp option, which allows partition-specific rates, 500 search replicates, and other parameters set to defaults. Clade support was assessed using 1000 replicates of the ultrafast bootstrap and an SH-aLRT test with 1000 replicates. Nodes with support values of both UFB ≥ 95 and SH-aLRT ≥ 80 are considered well supported (Nguyen et al. 2015), nodes with one of UFB < 95 or SH-aLRT < 80 are considered weakly supported, and nodes with both UFB < 95 or SH-aLRT < 80 are considered unsupported.

### Phylogenetic results

**Figure 1.**
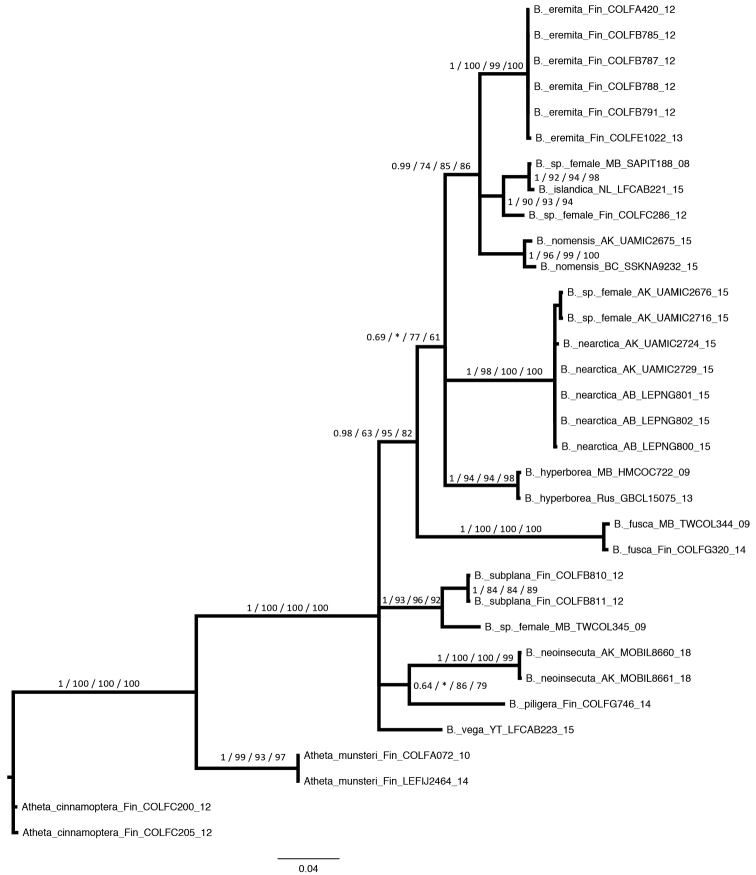
Fifty percent majority rule consensus phylogram from the Bayesian analysis with branch support values provided from left to right as: estimated posterior probabilities, maximum likelihood bootstrap proportions, ultrafast bootstrap values, and an SH-aLRT test values, with * = bootstrap values below 50%. Taxon identity is indicated for each sequence, followed by abbreviations of locality, and BOLD process IDs (see Table 1).

The resulting estimate of the mtDNA gene tree (Fig. 1) was relatively well resolved although a few relationships were obscured by polytomies or ambiguous due to low branch support values. The genus *Boreophilia*, as defined herein via morphology, was strongly supported as monophyletic (PP = 1.0, BS = 100, UFB = 100, SH-aLRT = 100). Notably, the species we transferred to *Boreophilia* (*B.piligera*) from *Boreostiba* was recovered within the clade of other *Boreophilia* while a species we transferred out of *Boreophilia* and into *Atheta* (*A.munsteri*) was recovered as the closest lineage to *Boreophilia*, with a long branch separating the two clades, thus supporting its exclusion from *Boreophilia*. Zero of the sampled Bayesian trees had *A.munsteri* nested within the *Boreophilia* clade, thus failing to reject the hypothesis that it is not a *Boreophilia* as morphologically defined herein. The *fusca* species group was supported as monophyletic with a strong posterior probability (0.98), ultrafast bootstrap (95%) and SH-aLRT support (82%) but relatively weak maximum likelihood bootstrap support (63%). The *subplana* species group, however, was not recovered as monophyletic due to its members and the *fusca* group emerging from a polytomy. Given the small size of the dataset, the *subplana* species group hypothesis remains ambiguous. All species with multiple specimens sampled were recovered as monophyletic with strong support (PP = 0.99 – 1.0, BS = 78–99% UFB = 84–100%, SH-aLRT = 89–100%) including two species, *B.hyperborea* and *B.fusca*, with samples from both the Nearctic and Palearctic. Nine of the ten species in our analysis are in BINs on BOLD (Table 1) with no species occurring in more than one BIN, and with no BIN holding more than one morphologically identified species.

Given the relatively small size of the dataset, in both taxon sampling and genetic data, we refrain from drawing any biogeographic conclusions based on these preliminary phylogenetic analyses. Additional genes including nuclear markers, greater specimen sampling within species, and addition of the missing *Boreophilia* species, would greatly improve our understanding of the evolution of these taxa.

Five specimens were female and could not be identified with certainty based on morphology alone (Fig. 1). Two of these (*B.* sp. TWCOL345_09 from Manitoba, Canada, and *B.* sp. COLFC286_12 from Finland) show branch lengths large enough to potentially be unique species not already represented in our dataset. The remaining females, (*B.* sp. SAPIT188_08, from Manitoba, and *B.* sp. UAMIC2676_15 and *B.* sp. UAMIC2716_15, from Alaska) cluster with sequences obtained from specimens identified via morphology, indicating the Manitoba female is likely *B.islandica* and the two Alaskan females are likely *B.nearctica*. Further comments relevant to the phylogenetic results and each species’ corresponding Barcode Index Numbers (BINs, Ratnasingham and Hebert 2013) on BOLD are listed under each species below.

### Summary of DNA distances within and among species

A full spreadsheet of DNA distances and our calculations is archived at https://doi.org/10.6084/m9.figshare.7822508. We summarize the key findings here. Among *Boreophilia* species, the minimum uncorrected ‘p’ distance within a species (limited only to sequences identified to species via morphology) in our dataset was 0.00%, the mean within species distance was 0.280%, and the maximum within species distance was 1.072%. Surprisingly, this maximum distance was found between two Nearctic samples (*B.nomensis* from British Columbia versus *B.nomensis* from Alaska) rather than between Palearctic versus Nearctic conspecific samples (*B.fusca* from Finland versus *B.fusca* from Manitoba, Canada were 0.539% distant and *B.hyperborea* from Russia versus *B.hyperborea* from Manitoba, Canada were 0.155% distant). This maximum within species distance is not a result of one of these sequences being incomplete (the British Columbia sequence is only 564 bp long while the Alaska sequence is 658 bp long). When these two sequences were compared after excluding base pairs missing from the shorter sequence, so both were 564 bp long, their distance was 1.064%, which remains the maximum within species value.

The minimum among species distance was 4.589% (between *B.nomensis* from Alaska and *B.eremita* from Finland), a value more than four times larger than the maximum within species distance. The average distance among species was 8.436%, and the maximum distance among species of *Boreophilia* was 12.080% (between *B.fusca* from Finland and *B.nearctica* from Alaska).

## Taxonomic review

### Tribe Athetini Casey, 1910

(sensu Klimaszewski et al. 2018)

#### Genus Boreophilia

Taxon classificationAnimaliaColeopteraStaphylinidae

Benick, 1973

Figs 1 , 2–5 , 6–15 , 16–23 , 24–31 , 32–42 , 43–52 , 53–56 , 57–62 , 63–70 , 71–80 , 81–83 , 84–91 , 92–99 , 100–108 , 109–116 , 117–119

Boreophilia Benick, 1973: 211; Lohse et al. 1990: 151; Gusarov 2003a, b: 81; Smetana 2004: 396; Schülke and Smetana 2015: 557; Klimaszewski et al. 2018. Type species: Homalotaislandica Kraatz, 1857.

##### Diagnosis.

*Boreophilia* may be distinguished from other athetines by the following combination of characters: body moderately narrow to broad, subparallel (Figs 6, 16, 24, 32, 43, 53, 57, 63, 71, 81, 84, 92, 100, 109, 117); head narrower or nearly as broad as pronotum (Fig. 6, 16, 24, 32, 43, 53, 57, 63, 71, 81, 84, 92, 100, 109, 117); eyes moderate in size, as long as or shorter than postocular region of head, tempora partially feebly carinate at base of head; antennae often reaching posterior margin of elytra, antennomeres V-X subquadrate, slightly transverse, or elongate (Figs 6, 16, 24, 32, 43, 53, 57, 63, 71, 81, 84, 92, 100, 109, 117); ligula divided into two separate and diverging lobes (Fig. 2); mandibles simple (not bifid at apex as in *Schistoglossa*) (Figs 3, 4); maxillary palps with 4 palpomeres, terminal one needle-shaped (Fig. 5); pronotum transverse, broadest in the middle or in apical fourth, hind angles rounded, hypomeron clearly visible in lateral view; integument smooth and moderately glossy, weak microsculpture present, punctuation fine, pubescence on midline of disc directed anteriad at least in apical ¾, and elsewhere directed laterally; mesocoxae contiguous; median lobe of aedeagus broad, flattened latero-ventrally, bulbus enlarged and tubus narrow, short or moderately elongate, approximately triangularly shaped in dorsal view (Figs 9, 10, 18, 26, 35, 36, 46, 59, 60, 65, 74, 83, 86, 94, 102, 103, 111), crista apicalis narrowly elongate in most, internal sac with two large, elongate sclerites in bulbus, and additional smaller sclerites in apical part of internal sac; male tergite VIII entire, and not modified (Figs 11, 19, 27, 37, 47, 61, 66, 75, 87, 95, 104, 112); spermatheca diverse in shape, with elongate tubular capsule and sinuate stem, invagination of capsule small or absent (Figs 15, 23, 31, 41, 42, 51, 52, 56, 70, 79, 80, 91, 99, 108, 116, 120); cold loving species occurring in arctic habitats, in temperate regions usually confined to fens and bogs.

**Figures 2–5.**
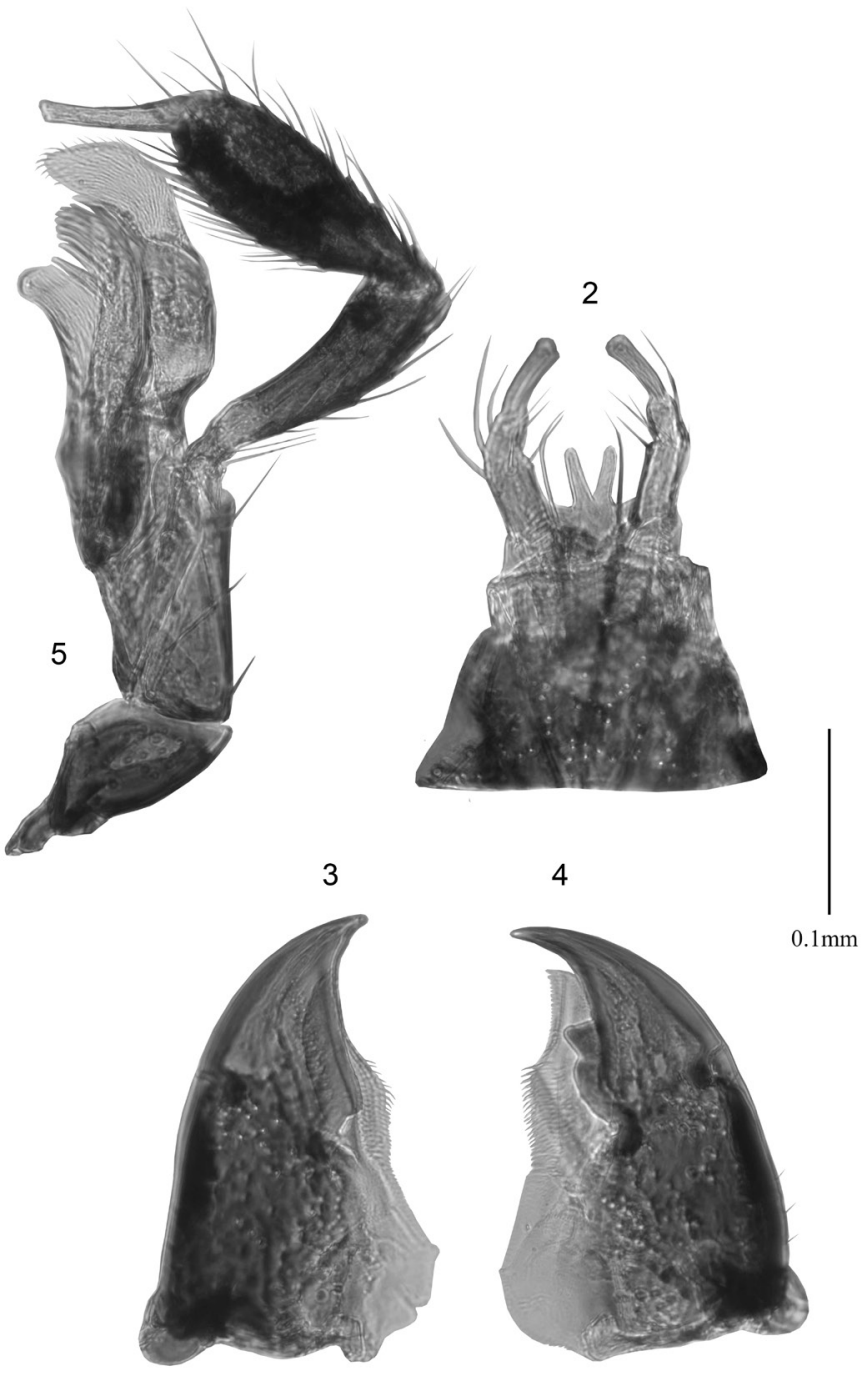
*Boreophiliaislandica* (Kraatz), mouthparts: **2** mentum, labial palps and ligula **3, 4** mandibles **5** maxilla. Scale bar: 0.1 mm.

#### Checklist of species occurring in the Nearctic Region

(species list follows that in the text, synonyms indented, see Schülke and Smetana 2015 for strictly Palaearctic synonyms)

1. *Boreophiliaeremita* (Rye, 1866). Fenyes 1920 (syn. of *Athetaislandica*); Lohse et al. 1990; Gusarov 2003a; Smetana 2004; Webster et al. 2012; Schülke and Smetana 2015; Klimaszewski et al. 2011, 2018.

**Holarctic species** (Distribution: north and central Europe, Ireland, Ukraine, Russia – West and East Siberia and the Far East; Canada: LB, NB, MB; USA: AK).

2. *Boreophiliaislandica* (Kraatz, 1857). Gusarov 2003; Smetana 2004; Klimaszewski et al. 2011, 2018.

**Holarctic species** (Distribution: Fennoscandia, Estonia, Faeroe Islands, Great Britain, Iceland, Russia – North European Territory; Canada: LB, NF, NT, NU, YT; USA: AK).

3. *Boreophiliafusca* (CR Sahlberg, 1831). Bernhauer, 1909; Lohse et al. 1990; Gusarov 2003a; Smetana 2004; Schülke and Smetana 2015.

**Holarctic species** (Distribution: Fennoscandia, Russia - North European Territory, West and East Siberia and the Far East; Canada: NT; USA: AK)

4. *Boreophiliahyperborea* (Brundin, 1940). Lohse et al. 1990; Gusarov 2003b; Smetana 2004; Ernst and Buddle 2013; Schülke and Smetana 2015.

**Holarctic species** (Distribution: Fennoscandia, Greenland, Russia – North European Territory; Canada: NT, NU; USA: AK)

5. *Boreophilianearctica* Lohse, in Lohse et al. 1990. Gusarov 2003b; Klimaszewski et al. 2011, 2018.

**Nearctic species** (Distribution: Canada: LB, QC, MB, AB [new record], YT, NF; USA: AK).

6. *Boreophiliaovalis* Klimaszewski and Langor, in Klimaszewski et al. 2011. Klimaszewski et al. 2018.

**Nearctic species** (Distribution: Canada: NF; USA: not recorded).

7. *Boreophilianomensis* (Casey, 1910). Gusarov 2003a.

**Nearctic species** (Distribution: Canada: YT, BC [new record]; USA: AK).

*Boreophiliacaseyiana* Lohse, in Lohse et al. 1990. Gusarov 2003a (synonym of *B.nomensis*).

8. *Boreophiliaventi* (Lohse), in Lohse et al. 1990.

**Nearctic species** (Canada: YT; USA: AK [new record]).

9. *Boreophilianeoinsecuta* Klimaszewski, sp. n. Misidentified in Lohse et al. 1990 (as *B.insecuta*).

**Nearctic species** (Distribution: Canada: MB, YT; USA: AK).

10. *Boreophiliaberingi* Klimaszewski & Brunke, sp. n.

**Nearctic species** (Distribution: USA: AK).

11. *Boreophiliasubplana* (J Sahlberg, 1880). Brundin 1954; Lohse et al. 1990; Gusarov 2003a; Smetana 2004.

**Holarctic species** (Distribution: Spitsbergen, Fennoscandia, Russia - West and East Siberia; Canada: NT, NU; USA: AK, NH).

*Boreophiliaangusticornis* (Bernhauer, 1907). Gusarov 2003a. **New synonymy**.

*Boreophiliaplutonica* (Casey, 1910). Gusarov 2003a: 83 (synonymy with *B.angusticornis*).

12. *Boreophiliacaseyi* Lohse, in Lohse et al. 1990, Gusarov 2003b.

**Nearctic species** (Distribution: Canada: MB, NU, YT; USA: AK).

*Boreophiliamanitobensis* Lohse, 1990, in Lohse et al. 1990. **New synonymy**.

(Distribution: Canada: MB; USA: AK)

13. *Boreophiliavega* (Fenyes, 1920, as *Atheta*). Smetana 2004; Schülke and Smetana 2015.

**Holarctic species** (Distribution: Russia - West and East Siberia, Far East, North Korea, Canada: YT; USA: not recorded).

*Boreophiliaherschelensis* Klimaszewski & Godin, 2012, in Klimaszewski et al. 2012. **New synonymy**.

14. *Boreophiliadavidgei* Klimaszewski & Godin, in Klimaszewski et al. 2012.

**Nearctic species** (Distribution: Canada: YT; USA: not recorded)

#### Species excluded from the genus *Boreophilia*

15. *Schistoglossablatchleyi* (Bernhauer & Scheerpeltz, 1926) (replacement name for *Athetacaviceps*Blatchley 1910 nec Poppius 1908). Gusarov 2003a (as *Atheta*); Klimaszewski et al. 2009 (transferred to *Schistoglossa*).

**Nearctic species** (Distribution: Canada: MB, NB, NWT, YT; USA: AK, IN).

*Boreophiliachillcotti* Lohse, in Lohse et al. 1990. Gusarov 2003 (synonym of *S.blatchleyi*). Synonymy confirmed here.

(Distribution: Canada: MB; USA: not recorded)

16. Atheta (Dimetrota) gelida J Sahlberg, 1887.Lohse et al. 1990 (as *Boreophilia*); Smetana 2004 (as *Boreophilia*); Schülke and Smetana 2015 (as *Boreophilia*); Klimaszewski et al. 2018 (as *Boreophilia*). Transferred back to Atheta here, subgenus Dimetrota, on basis of morphology of median lobe of aedeagus and spermatheca.

**Holarctic species** (Distribution: Russia: Chukotka Peninsula; Canada: MB, NWT, QC, YT; USA: AK)

17. Atheta (Dimetrota) munsteri Bernhauer, 1902. Lohse et al. 1990 (as Dimetrota); Smetana 2004 (as *Boreophilia*); Bousquet et al. 2013 (as Atheta (Dimetrota)); Schülke and Smetana 2015 (as *Boreophilia*).

**Holarctic species** (Distribution: northern Europe, North Korea; Canada: MB, NT, YT; USA: AK)

#### Key to species of *Boreophilia* recorded from the Nearctic region

**Table:** 

1	Body moderately narrow, elytra at base ca. as broad as maximum width of pronotum (Figs 6, 57, 71, 84)	**2**
–	Body broad, elytra at base distinctly broader than maximum width of pronotum (Figs 16, 24, 32, 43, 53, 63, 92, 100, 109, 117)	**5**
2	Elytra at suture shorter than pronotum at midline (Figs 57, 84)	**3**
–	Elytra at suture as long as or longer than pronotum at midline (Figs 6, 71)	**4**
3	Legs moderately long, hind legs much shorter that abdomen (Fig. 57); median lobe of aedeagus as illustrated (Figs 58–60), female unknown	***Boreophilianomensis* (Casey)**
–	Legs extremely long, hind legs almost as long as abdomen (Fig. 84); median lobe of aedeagus and spermatheca as illustrated (Figs 85, 86, 91)	***Boreophiliaberingi* Klimaszewski & Brunke, sp. n.**
4	Tubus of median lobe of aedeagus without basal projection on each side in dorsal view (Fig. 74); capsule of spermatheca club-shaped and with small apical invagination, stem broadly looped posteriorly (Figs 79, 80)	***Boreophilianeoinsecuta* Klimaszewski, sp. n.**
–	Tubus of median lobe of aedeagus with two basal projections on each side in dorsal view (Figs 9, 10); capsule of spermatheca elongate, tubular, and tapering apically, without apical invagination, stem narrowly looped posteriorly (Fig. 15)	***Boreophiliaeremita* (Rey)**
5	Maximum width of elytra one-fourth wider than pronotum (Figs 63, 100)	**6**
–	Maximum width of elytra one-fifth wider than pronotum (Figs 16, 24, 32, 43, 53, 92, 109, 117)	**7**
6	Median lobe of aedeagus with ventral margin of tubus arcuate basally and apex broad and rounded in lateral view (Fig. 64); sclerites of internal sac broad, curved and with small median projection in lateral view (Fig. 64); capsule of spermatheca narrowly club-shaped, spherical apically, stem coiled posteriorly (Fig. 70)	***Boreophiliaventi* (Lohse)**
–	Median lobe of aedeagus with ventral margin of tubus straight basally and apex broad and angular in lateral view (Fig. 101); sclerites of internal sac long, narrow and straight, without small median projection in lateral view (Fig. 101); capsule of spermatheca broadly club-shaped, hemispherical apically, stem hooked posteriorly (Fig. 108)	***Boreophiliacaseyi* Lohse**
7	Antennomeres VIII-X slightly to strongly elongate, less so in females (Figs 43, 53, 92)	**8**
–	Antennomeres VIII-X subquadrate to slightly transverse (Figs 16, 32, 109, 117)	**10**
8	Body broadly oval, robust, flattened (Fig. 53); antennomeres VIII–X at least one-fourth longer than maximum width (Fig. 53); female sternite VIII rounded apically with strongly sinuate antecostal line (Fig. 55); capsule of spermatheca narrowed apically and slightly pointed laterad; stem short, strongly sinuate and looped posteriorly (Fig. 56)	***Boreophiliaovalis* Klimaszewski & Langor**
–	Body not as above (Figs 43, 92); antennomeres VIII–X less elongate; female sternite VIII and spermatheca not as above	**9**
9	Median lobe of aedeagus narrow apically and slightly pointed in lateral view (Figs 44, 45); female sternite VIII truncate apically and slightly emarginate medially (Fig. 50); capsule of spermatheca evenly broad apically and evenly rounded at apex, stem broadly sinuate without posterior loop (Figs 51, 52)	***Boreophilianearctica* Lohse**
–	Median lobe of aedeagus broadly rounded apically in lateral view (Fig. 93), bulbus oval and broad apically in dorsal view (Fig. 94); sclerites of internal sac narrow (Figs 93, 94); capsule of spermatheca subspherical apically and apex rounded, stem narrow, sinuate, and narrowly looped posteriorly (Fig. 99)	***Boreophiliasubplana* (J Sahlberg)**
10	Pronotum width to length ratio 1.3 (Figs 32, 109, 117)	**11**
–	Pronotum width to length ratio 1.4–1.5 (Figs 16, 24)	**13**
11	Median lobe of aedeagus narrowly rounded apically in lateral view (Figs 33, 34), bulbus oval and narrowed apically in dorsal view (Figs 35, 36); sclerites of internal sac narrow (Figs 34, 36); capsule of spermatheca evenly broad apically, apex rounded, stem broad, sinuate, and looped posteriorly (Figs 41, 42)	***Boreophiliahyperborea* (Brundin)**
–	Genitalic structures not as above	**12**
12	Capsule of spermatheca with apical part ovoid, apical invagination not apparent, stem narrow and hooked posteriorly (Fig. 120); male undescribed	***Boreophiliadavidgei* Klimaszewski & Godin**
–	Capsule of spermatheca with apical part spherical, apical invagination present, stem moderately wide (Fig. 116); median lobe of aedeagus as illustrated (Figs 110, 111)	***Boreophiliavega* (Fenyes)**
13	Tubus of median lobe of aedeagus with rounded baso-lateral projection in lateral view (Fig. 17); capsule of spermatheca with narrowly rounded apical part (Fig. 23)	***Boreophiliaislandica* (Kraatz)**
–	Tubus of median lobe of aedeagus without basal projections in lateral view (Fig. 25); capsule of spermatheca with apical part narrowed and pointed laterad (Fig. 31)	***Boreophiliafusca* (CR Sahlberg)**

#### *Boreophiliafusca* species group [modified from Lohse et al. 1990]

This group contains mostly broad and large species (except *B.nomensis*), and is defined based on similarity of median lobe of aedeagus and spermatheca. Bulbus of aedeagus is moderately broad to broad, oval in shaped in dorsal view, with two prominent and elongate structures bearing ventrally a short, angular projection (Figs 9, 10, 18, 26, 35, 36, 46, 60); tubus of median lobe is triangular in dorsal view (Figs 9, 10, 18, 26, 35, 36, 46, 60), and in lateral view arcuate or straight and in most species narrow apically (Figs 7, 8, 17, 25, 33, 34, 44, 45, 58). Spermatheca: capsule pitcher shaped basally and tubular apically, apical invagination lacking; stem long, strongly sinuate and looped or arcuate posteriorly (Figs 15, 23, 31, 41, 42, 51, 52, 56).

**Species included**: *Boreophiliaeremita* (Rye), *B.islandica* (Kraatz), *B.fusca* (CR Sahlberg), *B.hyperborea* (Brundin), *B.nearctica* Lohse, *B.nomensis* (Casey), and *B.ovalis* Klimaszewski & Langor.

##### 1. Boreophilia eremita

Taxon classificationAnimaliaColeopteraStaphylinidae

(Rye, 1866)

Figs 6–15

Homalota eremita Rye, 1866: 123. Brundin 1953: 407 [as B.hercynica], Palm 1970: 260 [as B.hercynica], Lohse et al. 1990: 155, Smetana 2004: 396, Klimaszewski et al. 2011: 184, 2018: 566.
Atheta aluvialis Renkonen, 1936: 117. Smetana 2004: 396. Cotypus. Finland: Muonio, Renkonen, A.islandica Kr., var. alluvialis Renk (MHNG) 1 male, 1 female.
Atheta smolkai Rybiński, 1902: 11. Smetana 2004: 396.

###### Diagnosis.

**Figures 6–15.**
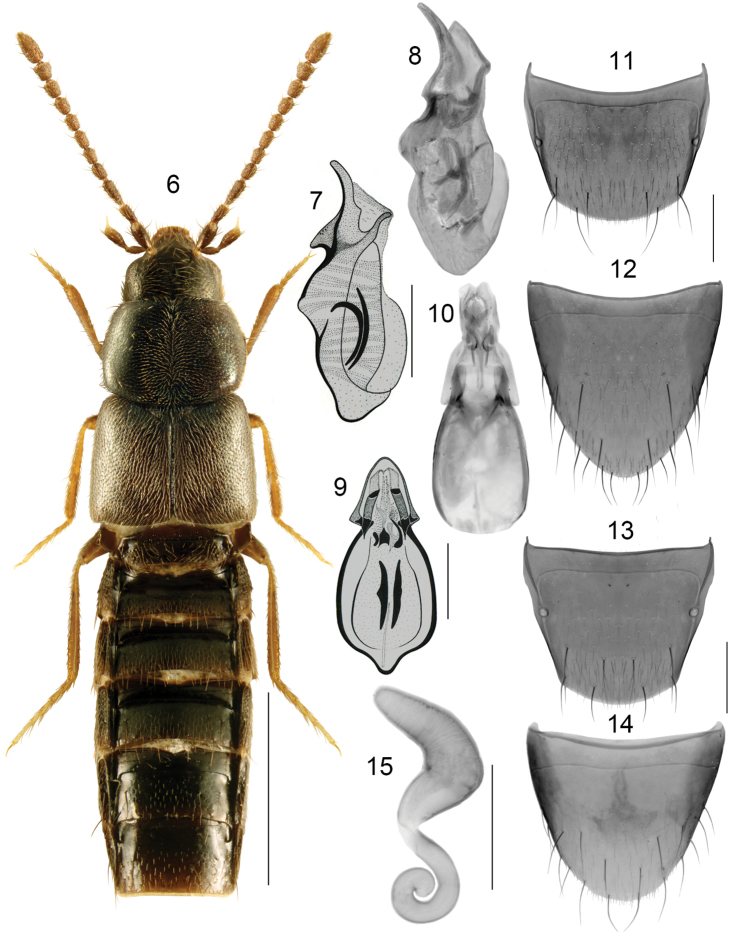
*Boreophiliaeremita* (Rye): **6** habitus **7, 8** median lobe of aedeagus in lateral view **9, 10** median lobe of aedeagus in dorsal view **11** male tergite VIII **12** male sternite VIII **13** female tergite VIII **14** female sternite VIII **15** spermatheca. Scale bars: 1 mm (for habitus); 0.2 mm (remaining).

Body moderately broad, forebody slightly and abdomen strongly glossy (Fig. 6); length 3.0–3.6 mm; dark brown to almost black, appendages light brown or reddish brown; antennomeres VIII-X subquadrate to slightly elongate; pronotum as long as elytra at suture, maximum width of pronotum slightly less than maximum width of elytra, pronotum in males is longer and less transverse that in females. **Male**. Tubus of median lobe of aedeagus with two, sharp basolateral projections on both sides in dorsal view (Figs 9, 10), and in lateral view ventral part of tubus produced angularly at base (Figs 7, 8), bulbus broadly oval in dorsal view, with two elongate narrow sclerites of internal sac (Fig. 9); tergite VIII arcuate apically (Fig. 11); sternite VIII elongate, parabolic apically (Fig. 12). **Female**. Spermatheca: capsule pitcher-shaped basally with elongate narrowly conical apical part without apical invagination, stem sinuate, narrowly looped posteriorly (Fig. 15); tergite VIII arcuate apically (Fig. 13); sternite VIII rounded apically, antecostal suture moderately sinuate (Fig. 14).

The median lobe of aedeagus of *B.eremita* is similar to that of *B.islandica*, but tubus is more elongate, narrower, less arcuate, and with basal projection angular in lateral view (Figs 7, 8), in dorsal view tubus with two pointed latero-basal projections (Figs 9, 10). In *B.islandica*, the tubus is broader, shorter, more arcuate ventrally, and with basal arcuate projection rounded in lateral view (Fig. 17), in dorsal view tubus with two smaller and less angular basal projections (Fig. 18). Spermathecae of the two species is variable in shape and very similar, female tergite and sternite VIII are similar in shape but sternite VIII in *B.eremita* has feebly arcuate medial part of antecostal suture (Fig. 14), which is strongly sinuate in *B.islandica* in the majority of examined specimens (Fig. 22). On average, the body of *B.eremita* is narrower and elytra shorter than that of *B.islandica*.

###### Distribution.

Holarctic species; recorded from north and central Europe, Ireland, Ukraine, Russia (west and east Siberia) and the Russian Far East; Canada: LB, NB, MB; USA: AK.

###### Collection data.

Habitat: in NB – old silver maple forest with green ash and seasonally flooded marsh; silver maple swamp, margin of vernal pond, found in moist leaves. In AK - creekside/ocean beach confluence, under boards and drift wood; black and white spruce, willow; subalpine habitat with *Veratrum*, and *Calamagrositis*. Collecting methods: sifting litter, Lindgren funnel trap, Malaise trap, hand collecting. Collecting period: May to August.

###### Additional material examined.

**NEARCTIC: Canada**, New Brunswick, Queens Co., Grand Lake Meadows P.N.A., 45.8227N, 66.1209W, 19–31.V.2010, old Silver Maple forest with Green Ash and seasonally flooded marsh, Lindgren funnel trap (LFC) 1 male; New Brunswick, Queens Co., Canning Grand Lake near Scotchtown, 45.8762N, 66.1817W, 25.V.2006, Silver maple swamp, near lake margin, margin of vernal pond in moist leaves, RP Webster coll. (LFC) 1 female.

**USA**, Alaska, mi 110 Denali Hwy., Seattle Creek, 15.VII.1978, JM Campbell and S Smetana, coll. GA Lohse MHNG-1994 (MHNG) 1 male.

**Females without male association, tentatively included in *B.eremita*** [they may represent *B.eremita* or extreme narrow forms of *B.islandica*]: **USA**, Alaska, Naknek, 58.73973N, 157.0636W, creekside/ocean beach confluence, under boards and drift wood, hand collected, 10.VI.2007, D. Sikes, UAM100012293, UAM100012313, UAM100012315 (UAM) 3 females; Kenai Pen., Kalifonsky Bch. Near Kenai, 8.VI.78, A Smetana and E Becker (MHNG) 1 female.

**PALEARCTIC: Finland**: Muonio, Renkonen (MHNG) 6 females. **Germany**: b. Grönau, Lübeck, 10.V.34, G Benick (MHNG) 1 female [ident. uncertain]. **Russia**: Siberia, Tschimilcan, FI. Lenam. [Lena River], B. Poppius (MHNG) 1 male.

###### Comments.

We have examined several European specimens identified as *B.hercynica*, which have the shape of median lobe of aedeagus and spermatheca similar to *B.eremita*, but the body color and the shape and proportions of forebody were different: body brown with dark brown head and pronotum and particularly elytra paler, and pronotum strongly transverse with sides broadly and evenly arcuate, elytra at suture slightly shorter than pronotum along midline. These specimens may represent extreme variation of *B.eremita* or a different and distinct species. Additional material is needed, and possibly DNA studies, to establish clear status of these specimens.

These specimens were labelled as follows: **Belgium**, Elsenborn, VIII.1931, Hohes Venn, coll. Benick (MHNG) 1 male; **Germany**, Brocken, Heinemann, *hercynica* s. mihi, det L Brundin (MHNG) 1 male; same label as before except: *islandica* Kr. (MHNG) 1 male; Brocken, Harz, 10.VI.1912, *islandica*, col. G Benick (MHNG) 1 male, Brocken, Heinemann (MHNG) 1 female, Brocken, 10–12.V.1913, Linke leg., *A.islandica* v. *hercynica* Renk., (MHNG) 1 female, Brockenkuppe, 26.VII.1916, coll. G Bennick (MHNG) 1 male; **Norway**, Kongberg [= Konsberg?], Münster, *islandica* Kr., coll. G Benick (MHNG) 1 female, Hammerfest, A Strand, *islandica* s. mihi, det. L Brundin (MHNG) 1 male, Norge, Skibotu, 22.VIII.1960, leg. Puthz, coll. Benick (MHNG) 1 male; **Scotland**, D Sharp, *hercynica* s. mihi det. L Brundin, *Metaxyaeremita* Rye (MHNG) 2 males; Shetland Is., Mainland, Stany Fields nr. Wal Is., 13.VII.1963, ME Bacchus BM 1963-471, coll. G Benick (MHNG) 1 female; **Sweden**, Dorotea, Sa. Lappland, 30–31.V.1935, Bruce, *A.islandica*, coll. Benick (MHNG) 1 female.

###### DNA Barcode data.

Our data included six sequences of *B.eremita* from Finland (four from Lapland and two from Northern Ostrobothnia), which grouped into BIN BOLD:ABW4331. BOLD reports these sequences have an average distance of 0.06%, a maximum distance of 0.18% and are 4.1% distant from their nearest neighbor.

##### 2. Boreophilia islandica

Taxon classificationAnimaliaColeopteraStaphylinidae

(Kraatz, 1857)

Figs 16–23

Homalota islandica Kraatz, 1857: 284. Smetana 2004: 396, Klimaszewski et al. 2011: 184, 2018: 567. **Syntypes**: Island, Krüper, coll. Kraatz, syntypus, islandica mihi (DEI) 1 male; 1 female. Examined.

###### Diagnosis.

**Figures 16–23.**
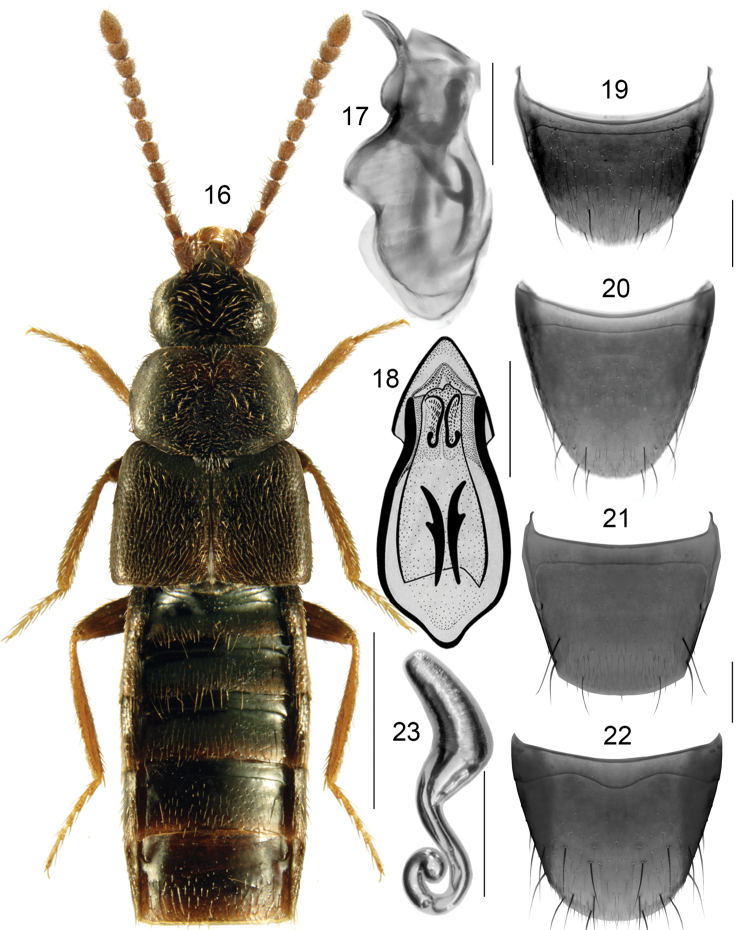
*Boreophiliaislandica* (Kraatz): **16** habitus **17** median lobe of aedeagus in lateral view **18** median lobe of aedeagus in dorsal view **19** male tergite VIII **20** male sternite VIII **21** female tergite VIII **22** female sternite VIII **23** spermatheca. Scale bars: 1 mm (habitus); 0.2 mm (remaining).

Body broad, forebody moderately and abdomen strongly glossy (Fig. 16); length 2.8–3.5 mm; uniformly black with paler, reddish brown appendages, or head, pronotum and VI-VII basal segments of abdomen dark brown, remainder of the body reddish brown, appendages light brown, sometimes elytra with some reddish tinge; antennomeres VIII-X subquadrate; pronotum as long as elytra at suture, maximum width of pronotum distinctly less than maximum width of elytra; elytral length variable, as long as pronotum or slightly longer. **Male**. Tubus of median lobe of aedeagus with two basolateral and slightly angular apically projections in dorsal view (Fig. 18), and one rounded projection in lateral view (Fig. 17); bulbus moderately broadly oval with two elongate sclerites of internal sac in dorsal view (Fig. 18); tergite VIII arcuate apically (Fig. 19); sternite VIII elongate, parabolic apically (Fig. 20). **Female**. Spermatheca: capsule pitcher-shaped basally with tubular apical projection moderately long and narrowed apically, stem coiled posteriorly, there is great variability in the shape of capsule (Fig. 23); tergite VIII arcuate apically (Fig. 21); sternite VIII rounded apically, antecostal suture strongly sinuate in most examined specimens (Fig. 22). For the differences between this and previous species see diagnosis of *B.eremita*.

###### Distribution.

Holarctic species; recorded from Fennoscandia, Estonia, Faeroe Islands, Great Britain, Iceland, Russia (North European Territory); Canada: LB, NF, NT, NU, YT; USA: AK.

###### Collection data.

Habitat [new data]: *Betula*, *Salix* litter; *Salix* tundra hillside; *Salix*/*Betula*/*Alnus*/grasses; black/white spruce, willow; vegetation at lakeshore pond; subalpine habitat with *Veratrum*, *Calamagrostis*, and *Leymus*, *Heracleum*, *Angelica*. Collecting methods: hanging Malaise trap, pitfall traps, sweeping with net. Collecting period: May to August.

###### Comments.

Females of this species may be confused with other species of *Boreophilia* and particularly those of closely related *B.eremita*. Associating females with males is considered here to be the most reliable way of identifying females of this and the previous species. At present, *B.islandica* is considered a somewhat variable species. Specimens vary from moderately robust and narrower to more robust and broader, with elytra as long as pronotum or slightly longer, all with the same morphology of genitalia. The BIN BOLD:ABX3767 formed a sister group to *B.islandica* in our analysis and was represented by a single female from Finland. The capsule of its spermatheca is curved at an angle of nearly 90 degrees and was among those shapes included in the illustrations of *B.islandica* by Palm (1970). BIN BOLD:ABX3767 may represent an undescribed species, or one of the described species not included in our DNA dataset, but corresponding males should be examined. A separate DNA based study is recommended to examine specimens of *B.islandica* sensu lato from a broad Holarctic distribution, including males and females.

###### Additional material examined.

**NEARCTIC: Canada**, Newfoundland, Long Range Mts., Portland Cr. Hill, 12–13.VIII.1982, Belland, Larson, McDonald (LFC) 1 female; Northwest Territories, 10.VI.1956, R.E. Leech (LFC) 1 male; Northwest Territories, Aklavik, 14.VI.1956, RE Leech (MHNG) 1 male, 1 female.

**USA**, Alaska: Anaktuvuk Pass, 647 m el., 68.14049N, 151.74091W, +/- 250 m, *Salix*, sweeping, 19.V.2016, D Sikes, K Daly, UAM100427773 (UAM) 1 male; Alaska, Aguttu Is., 15 m el., 52.473083N, 173.59065E, +/- 30 m, beach, *Heracl.lanatum*, 5 pitfalls, 5–19.VI.2008, L Kenney, R Kaler, UAM100029353 (UAM) 1 male; Aiktak Is., 10–20 m el., 54.18549N, 164.8432W, +/- 50 m, *Leymus*, *Heracleum*, maritime tundra, 5 pitfalls, 17–31.VIII.2009, A.L. Larned, UAM100321897, UAM100321780 (UAM) 2 males; same data except: *Leymus*, *Heracleum*, *Angelica*, 11–29.VII.2009, UAM100321769 (UAM) 1 female; UAM100321861 (UAM) 1 female; 54.18356N, 164.82793W, +/- 1 km, maritime tundra, *Leymus*, *Heracleum*, 5 pitfalls, 9–24.VI.2009, AL Larned, UAM100322546 (UAM) 1 female; 24.VI-11.VII.2009, S. Sapora, UAM100323153, UAM100323220 (UAM) 2 females; 54.11036N, 164.50500W, +/- 5 m, streamside, *Leymus*, 2 pitfalls, 26.VI-14.VII.2008, BA Drummond, UAM100030104, UAM100030117 (UAM) 2 females.

**Females without male association, tentatively included as *B.islandica***:

**USA**, Alaska: White Mtns. Rec. Area, 180 m el., 65.33469N, 146.83969W, +/- 10 m, b. & w. spruce, willow, hanging Malaise, 10–17.VI.2016, J Hagelin, UAM100407456 (UAM) 2 females; S. Chilkat Pen., pl. 19, 652 m el., 58.42509N, 135.14229W, +/- 30 m, subalpine, *Veratrum*, *Calamagrostis*, hand coll., 9.VII.2010, DS Sikes, UAM100277138 (UAM) 2 females; Kasatochi, 42 m el., 52.16869N, 175.52475W, +/- 34 m, under rocks, Talus, cliff base, 11.VI.2008, DS Sikes, UAM100031453, UAM100031445 (UAM) 2 females.

**PALEARCTIC: Czech Republic**: Bohemia occ., Frant. Láznê-Soos, 1961, Smetana (MHNG) 2 females. **Denmark**: Helsinge, 6.V.1915, Häkan Lindberg (MHNG) 1 female. **Finland**: Kittilä, Renkonen (MHNG) 1 female; Lapp., Petsamo, 3.VII.1929, Häkan Lindberg (MHNG) 1 female; Oa Lappfiärd, 3.V.1944, Harald Lindberg (MHNG) 1 female. **Germany**: I. b. Grönau, Lübeck, 27.III.12, G. Benick (MHNG) 1 female; Brocken, 15.V.32, Foln.…[not clear] (MHNG) 1 female; Brocken, Heineman (MHNG) 1 female; Brocken Harz, 15.VII.1935, Fehse, Thale, G Benick (MHNG) 1 female. **Island**: Island, VII.1969, G Benick (MHNG) 1 male. **Norvay**: Lakselv Po, A Strand (MHNG) 3 females. **Sweden**: Lule Lappmark, Vietas 20, 27.VI.1967, TE Leiler (MHNG) 1 female; Moen M. elev., A Strand (MHNG) 1 female; Ő Torne, T Palm (MHNG) 1 female; Imtl. Frost-viken, Palm (MHNG) 1 female.

###### DNA Barcode data.

Our data included one sequence identified as *B.islandica* from Newfoundland and Labrador, Canada, and one sequence identified as *Boreophilia* sp. collected from Churchill, Manitoba, which are the only members of BIN BOLD:AAH0226. BOLD reports these sequences are 2.79% distant from their nearest neighbor.

##### 3. Boreophilia fusca

Taxon classificationAnimaliaColeopteraStaphylinidae

(CR Sahlberg, 1831)

Figs 24–31

Aleochara fusca CR Sahlberg, 1831: 371. Lohse et al. 1990: 152, Smetana 2004: 396.

###### Diagnosis.

**Figures 24–31.**
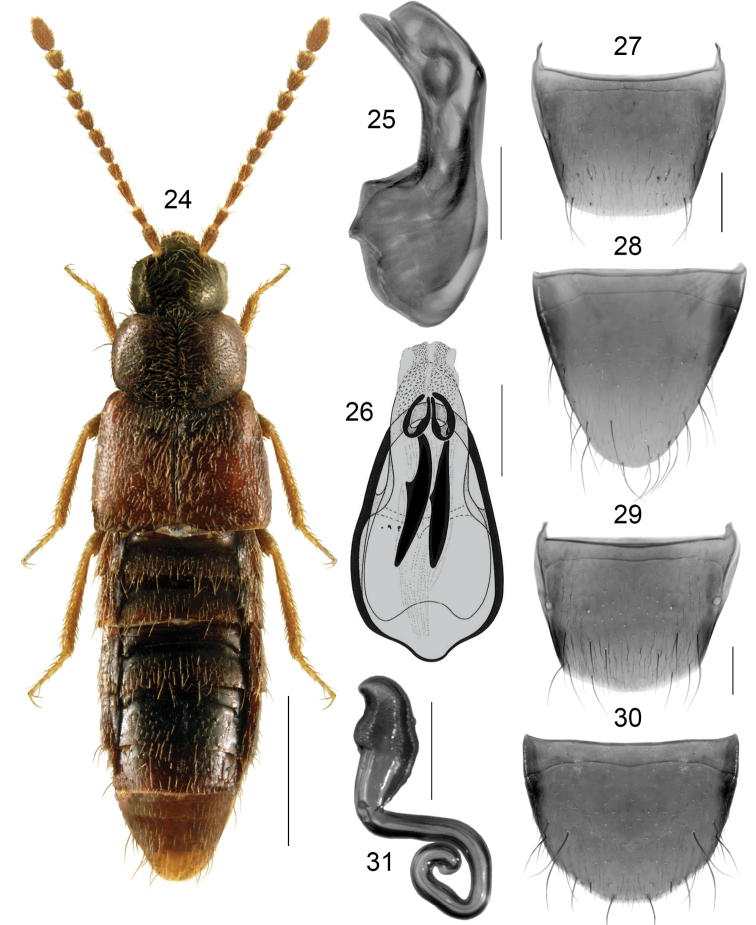
*Boreophiliafusca* (C.R. Sahlberg): **24** habitus **25** median lobe of aedeagus in lateral view **26** median lobe of aedeagus in dorsal view **27** male tergite VIII **28** male sternite VIII **29** female tergite VIII **30** female sternite VIII **31** spermatheca. Scale bars: 1 mm (for habitus); 2 mm (remaining).

Body broad, forebody moderately and abdomen slightly more glossy (Fig. 24); length 3.4–3.8 mm; head, pronotum and abdomen except for its apex dark brown, elytra dark-reddish brown, appendages light brown, or body entirely dark brown to almost black with tarsi reddish brown; antennomeres VIII-X subquadrate; pronotum shorter than elytra at suture, maximum width of pronotum distinctly less than maximum width of elytra. **Male**. Tubus of median lobe of aedeagus evenly arcuate laterally, apex narrow and slightly pointed in lateral view (Fig. 25), bulbus oval, broad basally and narrowed apically in dorsal view, and with two elongate sclerites of internal sac (Fig. 26); tergite VIII arcuate apically (Fig. 27); sternite VIII elongate, parabolic apically (Fig. 28). **Female**. Spermatheca: capsule pitcher-shaped basally with broadly tubular apical projection, moderately long and pointed apico-laterally, stem coiled posteriorly (Fig. 31); tergite VIII arcuate apically (Fig. 29); sternite VIII rounded apically, antecostal suture slightly sinuate (Fig. 30).

###### Distribution.

Holarctic species; recorded from Fennoscandia, Russia (west and east Siberia) and the Far East; Canada: NT; USA: AK.

###### Collection data.

Habitat: tundra. Collecting methods: not recorded in Nearctic region. Collecting period: June and July.

###### Additional material examined.

**NEARCTIC: Canada**, NT, Aklavik, 16.VI.1956, EF Cashmann, fusca Sahlb. Det. Lohse (MHNG) 1 male; NT, Aklavik, 29.VI.1956, EF Cashmann, fusca Sahlb. Det. Lohse (MHNG) 1 female.

**PALEARCTIC: Finland**: Muonio, Renkonen, 2531, *A.fusca* Sahlb., Renkonen det., coll. G Benick (MHNG) 2 females. Country unspecified: Bjerkeng Schn. 21.VI.1912, coll. G Benick (MHNG) 1 male.

###### DNA Barcode data.

Our data included two sequences of *B.fusca*, one from Finland and one from Manitoba, Canada, which grouped into BIN BOLD: AAG4311. BOLD reports these sequences have an average and maximum distance of 0.54% and are 9.68% distant from their nearest neighbor.

##### 4. Boreophilia hyperborea

Taxon classificationAnimaliaColeopteraStaphylinidae

(Brundin, 1940)

Figs 32–42

Atheta hyperborea Brundin, 1940: 131. Lohse et al. 1990: 153, Smetana 2004: 396.

###### Diagnosis.

**Figures 32–42.**
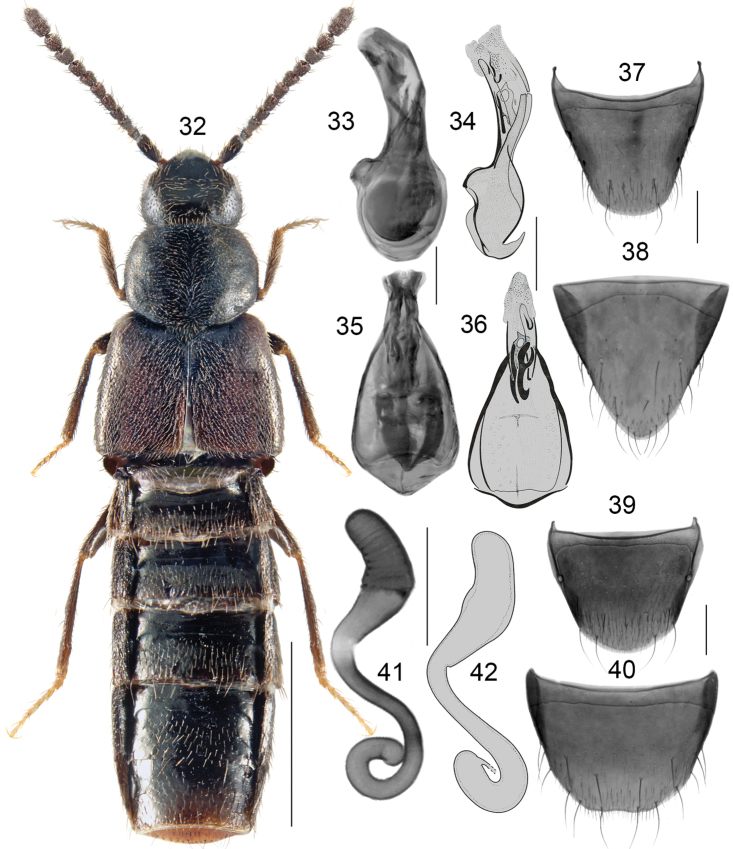
*Boreophiliahyperborea* (Brundin): **32** habitus **33, 34** median lobe of aedeagus in lateral view **35, 36** median lobe of aedeagus in dorsal view **37** male tergite VIII **38** male sternite VIII **39** female tergite VIII **40** female sternite VIII **41, 42** spermatheca. Scale bars: 1 mm (for habitus); 0.2 mm (remaining).

Body broad, forebody glossy; length 2.8–3.5 mm; black with tarsi reddish brown (Fig. 32); antennomeres VIII-X subquadrate; pronotum as long as or slightly shorter than elytra at suture, maximum width of pronotum distinctly less than maximum width of elytra. **Male**. Tubus of median lobe of aedeagus narrow, broadly arcuate in lateral view, apex narrow and rounded (Figs 33, 34), bulbus large, oval, broad basally and narrowed apically in dorsal view, and with two elongate sclerites (Figs 35, 36); tergite VIII arcuate apically (Fig. 37); sternite VIII elongate, parabolic apically (Fig. 38). **Female**. Spermatheca: capsule pitcher-shaped basally with evenly, broadly tubular apical projection, moderately long and rounded apically, stem sinuate, half-looped posteriorly (Figs 41, 42); tergite VIII broadly rounded apically (Fig. 39); sternite VIII rounded apically and truncate medially, antecostal suture straight medially and slightly sinuate laterally (Fig. 40).

###### Distribution.

Holarctic species; recorded from Fennoscandia, Greenland, Russia (North European Territory); Canada: NT, NU; USA: AK.

###### Collection data.

Habitat: tundra, under rocks. Collecting methods: hand collected from under rocks. Collecting period: June and July.

###### Additional material examined.

**NEARCTIC: Canada**, NT, Barthurst Inl., Hiukitak River, 3.VIII.1966, GE Shewell, *B.hyperborea* Brn., det. GS Lohse (NHNG) 1 male.

**USA**, Alaska, Toolik Field Station, 724 m el., 68.6286N, 149.59772W, +/- 36 m, under rocks, 1- 3.VI.2008, D.S. Sikes, UAM100031281 (UAM) 1 male; Anaktuvuk Pass, 665 m el., 68.14059N, 151.74266W, +/- 200 m, *Salix*, tundra hillside, cobble, pollinator pitfall, 20.V.2016, D. Sikes, K. Daly, UAM100427687 (UAM) 1 female [tentative association].

**PALEARCTIC: Norway**, Vaalaasjö Andr. Strand, coll. G. Benick (NHNG) 1 female; Barviksmyren, W of Smelror, Varangerhalvøya, 22.VII.1998, V. Mahler (UCC) 1 female.

**Greenland**. Sdr. Strømfjord, 1.VII.1979, Brundin det. 1940 (NHMD) 1 female.

###### DNA Barcode data.

Our data included two sequences of *B.hyperborea*, one from Russia and one from Manitoba, Canada, which grouped into BIN BOLD:AAG4302. BOLD reports these sequences have an average and maximum distance of 0.16% and are 6.82% distant from their nearest neighbor.

##### 5. Boreophilia nearctica

Taxon classificationAnimaliaColeopteraStaphylinidae

Lohse, 1990

Figs 43–52

Boreophilia nearctica Lohse, in Lohse et al. 1990: 153. Klimaszewski et al. 2011: 185, 2018: 567. **Paratype.** AK, mi 1252 Alaska Hwy., 7.VII.1968, nearctica nov. sp. Lohse, Paratype, Campbell and Smetana (NHNG) 1 male.

###### Diagnosis.

**Figures 43–52.**
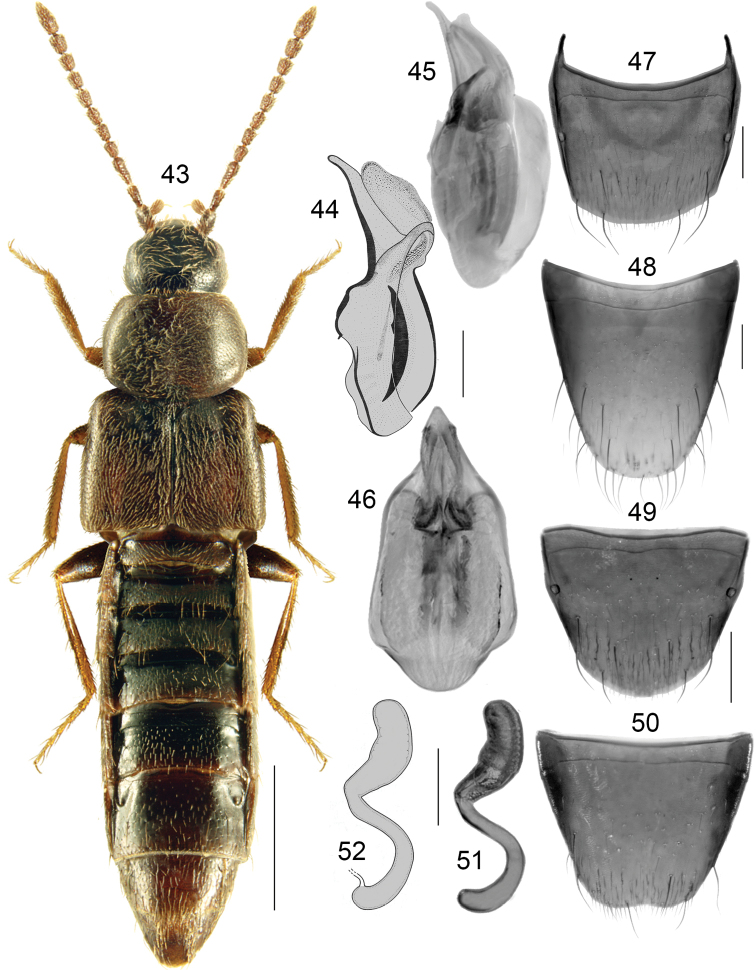
*Boreophilianearctica* Lohse: **43** habitus **44, 45** median lobe of aedeagus in lateral view **46** median lobe of aedeagus in dorsal view **47** male tergite VIII **48** male sternite VIII **49** female tergite VIII **50** female sternite VIII **51, 52** spermatheca. Scale bars: 1 mm (for habitus); 0.2 mm (remaining).

Body moderately broad, forebody moderately glossy, abdomen slightly more so (Fig. 43); length 3.0–3.5 mm; head, pronotum and abdomen except for its apex dark brown, elytra reddish brown, appendages light brown, or body dark brown to almost black with elytra partially dark reddish brown and tarsi reddish brown; antennomeres VIII-X subquadrate to slightly elongate; pronotum ca. as long as elytra at suture, maximum width of pronotum distinctly less than maximum width of elytra. **Male**. Tubus of median lobe of aedeagus almost straight ventrally, apex narrow and pointed, slightly produced ventrally in lateral view (Figs 44, 45), bulbus broadly oval in dorsal view and with two elongate sclerites of internal sac (Fig. 46); tergite VIII arcuate apically, slightly pointed medially (Fig. 47); sternite VIII elongate, parabolic apically (Fig. 48). **Female**. Spermatheca: capsule pitcher-shaped basally with broadly tubular apical projection, moderately long and rounded apically, stem sinuate and without posterior loop (Figs 51, 52); tergite VIII arcuate apically (Fig. 49); sternite VIII truncate apically and slightly emarginate medially, antecostal nearly straight medially and slightly sinuate laterally (Fig. 50).

###### Distribution.

Nearctic species; recorded from Canada: AB [**new record**], LB, MB, QC, YT, NF; USA: AK.

###### Collection data.

Habitat [new data]: black spruce forest; alpine meadow. Collecting methods: hanging Malaise trap, pitfall traps, hand collecting under rocks and litter. Collecting period: July to September.

###### Additional material examined.

**Canada**, Alberta, Plateau Mountain, 50.226 -114.555, alpine meadow, under rocks and litter, 5.VII.2002, G Pohl and D Langor, 2 males, 1 female (CCDB-28541-D04, CCDB-28541-D05, CCDB-28541-D06) (NFRC); Newfoundland, Burnt Cape, site 10, 21U 0586332E, 5711616N, 51.54902N, 5.75489W, 24.VII.2003–7.VIII.2003 (LFC) 1 male; Burnt Cape, 55.755W, 51.549W, Coastal meadow, pitfall trap, 10–24.VII.2003, AM Hynes, Site 1–10 (LFC) 1 male; Yukon Territory, North Fork Pass, Ogilvie Mts.,3500’, 17.VI.1962, RE Leech, paratype CNC No. 20308 (CNC) 1 male; North Fork Pass, Ogilvie Mts., 4100’, 20.VI.1962, PJ Skitsko (CNC) 1 female; Quebec, Bonne Esperance, 14.VII.1929, WJ Brown, *nearctica* nov. sp. Lohse, Paratype (NHNG) 1 female.

**USA**, Alaska: Naknek, 58.73973N, 157.0636W, creek side/ocean beach confluence, under boards and drift wood, hand collected, 10.VI.2007, DS Sikes, UAM100012316 (UAM) 1 male; AK, Selawik NWR, 8 m el., 66.56137N, 158.99834W, +/- 304 m, *Spirea*, dwarf birch, 23–24.VI.2010, DS Sikes, UAM100283949 (UAM) 1 male; AK, Fairbanks, Bonanza Crk., 237 m el., 64.71922N, 148.38416W, +/- 10 m, birch, spruce, hanging Malaise trap, 6–13.V.2016, J Hagelin, UAM100407183 (UAM) 1 male; AK, Kenai Mts., Tern Lake Campground, 700’, 18.Vi.1978, Smetana and Becker (NHNG) 1 male.

###### Comments.

The southernmost record of this species in the Rockies of southern Alberta suggests that *B.nearctica* probably occurs continuously along the western cordilleras, at successively higher elevation sites with decreasing latitude.

###### DNA Barcode data.

Our data included five sequences of specimens identified as *B.nearctica*, two from Alaska and three from Alberta, Canada, which grouped with two sequences identified as *Boreophilia* sp. into BIN BOLD:ACU9385. Our calculations indicate that the five sequences identified to species have an average distance of 0.14%, a maximum distance of 0.33% and are 6.37% distant from their nearest neighbor.

##### 6. Boreophilia ovalis

Taxon classificationAnimaliaColeopteraStaphylinidae

Klimaszewski & Langor, 2011

Figs 53–56

Boreophilia ovalis Klimaszewski & Langor, in Klimaszewski et al. 2011: 186. Klimaszewski et al. 2018: 568.

###### Diagnosis.

**Figures 53–56.**
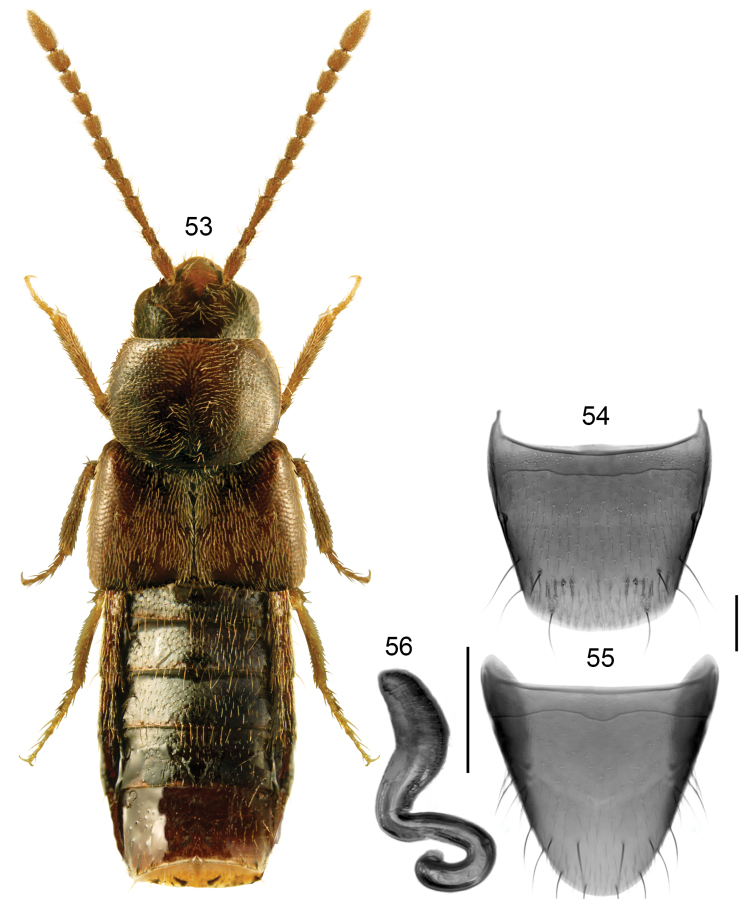
*Boreophiliaovalis* Klimaszewski and Langor: **53** habitus **54** female tergite VIII **55** female sternite VIII **56** spermatheca. Scale bars: 1 mm (for habitus); 0.2 mm (remaining).

Body very broad, forebody moderately and abdomen strongly glossy (Fig. 53); length 3.0–3.5 mm; head, pronotum and abdomen except for its apex dark brown, elytra dark-reddish brown medially, appendages brown, or entire body dark brown to almost black and tarsi reddish brown; antennomeres VIII-X elongate; pronotum ca. as long as elytra at suture, maximum width of pronotum slightly less that the maximum width of elytra. **Male**. Unknown. **Female**. Spermatheca: capsule pitcher-shaped basally with broadly tubular and slightly pointed apical part, stem short, strongly sinuate and looped posteriorly (Fig. 56); tergite VIII arcuate apically (Fig. 54); sternite VIII rounded apically, antecostal suture strongly sinuate medially (Fig. 55). Females of this species may be confused with those of *B.fusca*, from which they differ by distinctly elongate antennomeres VIII-X, more deeply medially sinuate antecostal suture of sternite VIII, and spermathecal capsule more evenly elongate and apex less pointed laterad.

###### Distribution.

Nearctic species, recorded only from Canada: NF.

###### Collection data.

Habitat: unspecified forest. Collecting methods: one female was captured in Malaise trap. Collecting period: June to September.

###### DNA Barcode data

. Two specimens of this species, one being a paratype, were submitted for DNA barcoding but failed to generate DNA sequences (process IDs on BOLD: LFCAB222-15, NGSFT931-15).

##### 7. Boreophilia nomensis

Taxon classificationAnimaliaColeopteraStaphylinidae

(Casey, 1910)

Figs 57–62

Dinaraea nomensis Casey, 1910: 96. As Boreophilia: Lohse et al. 1990: 160; Gusarov 2003: 81. **Lectotype** (male): USA, Alaska, Nome (USNM). Designated by Gusarov 2003: 81.
Boreophilia caseyiana Lohse 1990, in Lohse et al. 1990. Synonymized by Gusarov 2003. **Holotype** (male): Canada, Yukon Territory, North Fork Pass, Ogilvie Mts., 3500’, 17.VI.1962, RE Leech, CNC No. 20312 (CNC).

###### Diagnosis.

**Figures 57–62.**
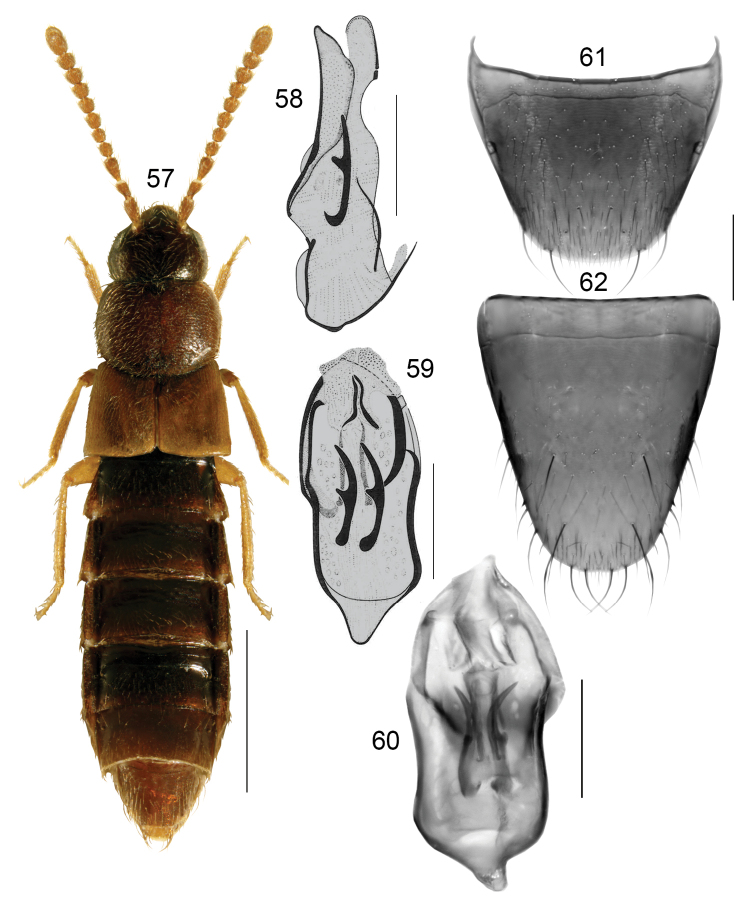
*Boreophilianomensis* (Casey): **57** habitus **58** median lobe of aedeagus in lateral view **59, 60** median lobe of edeagus in dorsal view **61** male tergite VIII **62** male sternite VIII. Scale bars: 1 mm (for habitus); 0.2 mm (remaining).

Body narrow, subparallel, moderately glossy, abdomen slightly more so (Fig. 57); length 3.0–3.8 mm; head, pronotum and abdomen dark brown, elytra reddish brown, legs paler, yellowish brown; antennomeres VIII–X subquadrate; pronotum longer than elytra at suture, maximum width of pronotum ca. the same as maximum width of elytra. **Male**. Tubus of median lobe of aedeagus almost straight (slightly arcuate) in lateral view, apex triangular in shape, slightly pointed (Fig. 58), bulbus broad in dorsal view and with two elongate narrow sclerites as illustrated (Figs 59, 60); tergite VIII arcuate apically (Fig. 61); sternite VIII elongate, parabolic apically (Fig. 62). **Female**. Undescribed.

###### Distribution.

Nearctic species, recorded from Canada: YT, BC [new record]; USA: AK.

###### Collection data.

Habitat: spruce and aspen forest with horsetail/shrub/grass undergrowth; edge of snowfield. Collecting methods: pitfall trap, hand collecting under rocks. Collecting period: June, July and August.

###### Additional material examined.

**Canada**, British Columbia, Kinaskan Lake Provincial Park, Kinaskan Lake Trail, 57.532–130.202, 833 m, spruce and aspen forest, pitfall trap, 1.VIII.2014, BIObus 2014, (BIOUG24477-H04) (CBG). **USA**, Alaska, Thompson Pass, 61.137 -145.745, under rocks nr. snowfield, 28.VII.2010, DS Sikes and AB Sikes (UAM100288002) (UAM).

###### DNA Barcode data.

Our data included two sequences of specimens identified as *B.nomensis*, one from Alaska and one from British Columbia, Canada which grouped into BIN BOLD:ACU9384. BOLD reports these sequences have an average and maximum distance of 1.06% and are 4.64% distant from their nearest neighbor.

#### *Boreophiliasubplana* species group

This newly defined group contains species defined by the similarity of the median lobe of aedeagus (Figs 64, 65, 72–74, 82, 83, 85, 86, 93, 94, 101–103, 110, 111), and capsule of spermatheca with a small apical invagination (Figs 70, 79, 80, 91, 99, 108, 116, 120). Bulbus of median lobe of aedeagus broad apically, oval in shape in dorsal view, with two prominent elongate structures of slightly different shape in different species (Figs 65, 74, 83, 86, 94, 102, 103, 111); tubus of median lobe of aedeagus arcuate or straight and broad or narrow in lateral view (Figs 64, 72, 73, 82, 85, 93, 101, 110); internal sac broadly open apically and supported by two narrow arcuate structures (Figs 65, 74, 83, 86, 94, 102, 103, 111). Spermatheca: capsule pitcher shaped basally and globular or ovoid apically with small apical invagination in most species; stem long strongly sinuate and looped or coiled posteriorly (Figs 70, 79, 80, 91, 99, 108, 116, 120).

**Species included**: *Boreophiliainsecuta* (Eppelsheim), *B.neoinsecuta* Klimaszewski, sp. n., *B.beringi* Klimaszewski & Brunke, sp. n., *B.subplana* (J. Sahlberg), *B.caseyi* Lohse, *B.vega* Fenyes, *B.venti* (Lohse), and *B.davidgei* Klimaszewski & Godin.

##### 8. Boreophilia venti

Taxon classificationAnimaliaColeopteraStaphylinidae

(Lohse, 1990)

Figs 63–70

Dimetrota venti (Lohse), in Lohse et al. 1990: 183.

###### Holotype (male).

Canada, Yukon Territory, British Mts., Windy Ridge, 450 m, 69.27N, 140.26W, 2.VII.1984, 84–47, sifting *Salix* litter, JM Campbell (CNC).

###### Paratypes.

labeled as the holotype (CNC) 3 sex undetermined.

###### Diagnosis.

**Figures 63–70.**
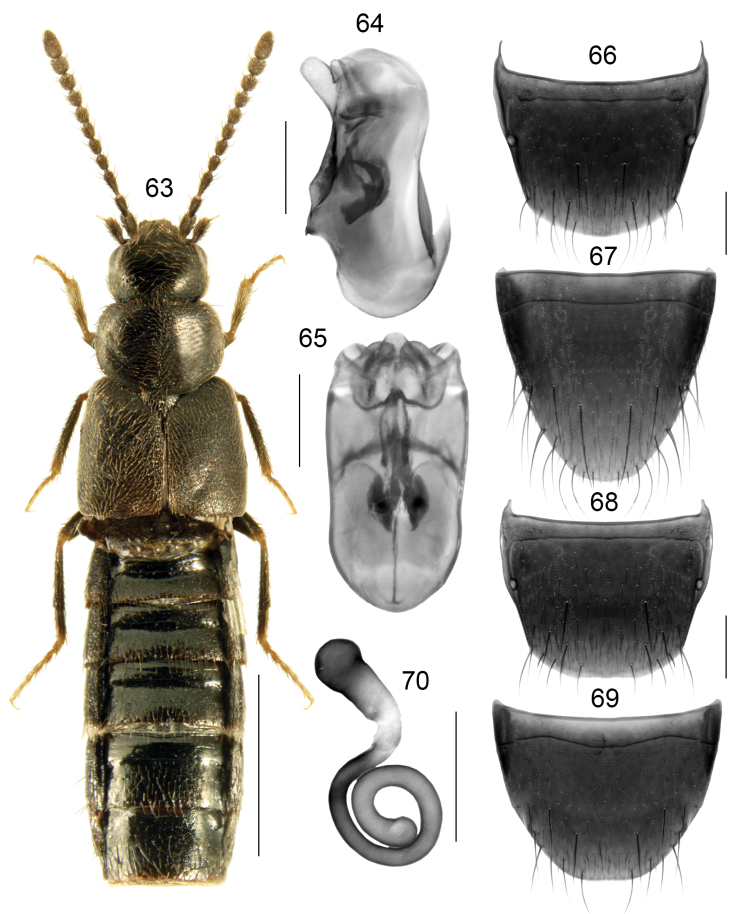
*Boreophiliaventi* (Lohse): **63** habitus **64** median lobe of aedeagus in lateral view **65** median lobe of aedeagus in dorsal view **66** male tergite VIII **67** male sternite VIII **68** female tergite VIII **69** female sternite VIII **70** spermatheca. Scale bars: 1 mm (for habitus); 0.2 mm (remaining).

Body narrowly subparallel, forebody moderately glossy, abdomen slightly more so (Fig. 63); length 3.0–3.4 mm; uniformly dark brown to black, appendages yellowish brown or reddish brown; antennomeres VIII-X subquadrate; pronotum ca. as long as elytra at suture, maximum width of pronotum slightly less than maximum width of elytra. **Male**. Tubus of median lobe of aedeagus arcuate basally, straight apically and produced ventrad in lateral view, apex broad and rounded in lateral view (Fig. 64), bulbus broad and with two large sclerites, each apically curved, sharply pointed, and with small median projection (Figs 64, 65); tergite VIII arcuate apically (Fig. 66); sternite VIII elongate, parabolic apically (Fig. 67). **Female**. Spermatheca: capsule club-shaped, tubular basally and rounded apically, with small apical invagination; stem sinuate and coiled posteriorly (Fig. 70); tergite VIII arcuate apically and truncate medially (Fig. 68); sternite VIII rounded apically and truncate medially, antecostal suture distinctly sinuate (Fig. 69). Spermatheca of this species is very similar to that of *B.neoinsecuta* (Figs 79, 80), but shape of apex of female sternite VIII are different in the two species (Figs 69, 78).

###### Distribution.

Holarctic species, recorded from Europe, Finland; Asia, East and West Siberia, Mongolia; and North America: Canada: YT; USA: AK [**new record**].

###### Collection data.

Habitat [new data]: *Salix* litter; *Salix* tundra hillside, lakeshore debris. Collecting methods: sifting *Salix* litter, pitfall traps, hand/aspirator collecting from under rocks. Collecting period: May and July.

###### Additional material examined.

**USA**, Alaska, Anaktuvuk Pass, 640–680 m el., 68.14049N, 151.74091W,+/- 2 km in and around village, hand, forceps, 2–22.V.2016, D Sikes, K Daly UAM100427681 (UAM) 1 female; Anaktuvuk Pass, 665 el., 68.14059N, 151.74266W, +/- 200 m, *Salix*, tundra hillside, cobble, pollinator pitfall, 20.V.2016, D Sikes, K Daly, UAM100427683 (UAM) 1 male; UAM100427684 (UAM) 1 female; UAM100427689 (UAM) 1 female; UAM100427693 (UAM) 1 male.

###### Comments.

Lohse, in Lohse et al. 1990, described *Dimetrotaventi* from Yukon. This species is extremely similar externally and genitally to *Boreophiliainsecuta* described from Europe. The genitalia of *B.insecuta* were illustrated by Brundin (1954), and here, based on a specimen from Siberia (Figs 82, 83).

In *B.venti*, the tubus of the median lobe is distinctly arcuate in lateral view (Fig. 64), but in *B.insecuta* it is always straight basally (Fig. 82) and the two main sclerites are slightly different in shape (Figs 64, 65, 82, 83). However, the remaining genital characters are similar and females of the two species are not distinguishable morphologically.

##### 9. Boreophilia neoinsecuta

Taxon classificationAnimaliaColeopteraStaphylinidae

Klimaszewski sp. n.

http://zoobank.org/364757AB-CD31-40E6-82E5-BEE1903B9621

Figs 71–80

Boreophilia insecuta sensu Lohse, in Lohse et al. 1990: 157. **Misidentification**.

###### Holotype.

(male): USA, Alaska, North Slope, Atkasuk, 17.VII.1978, B Vogel coll., B.insecuta det. Lohse (CNC).

###### Paratypes.

USA, Alaska, Anaktuvuk Pass, 647 m el., 68.14049N, 151.74091W, +/- 250 m, under rocks, forceps/aspirators, 19.V.2016, D Sikes, K Daly, UAM100413204, UAM100413205, UAM100413207 (UAM) 3 females; Anaktuvuk Pass, 640–680 m el., 68.14049N, 151.74091W, +/- 2 km in and around village, hand/forceps, 20–22.V.2016, D Sikes, K Daly, UAM100388381 (UAM) 1 female.

**Figures 71–80.**
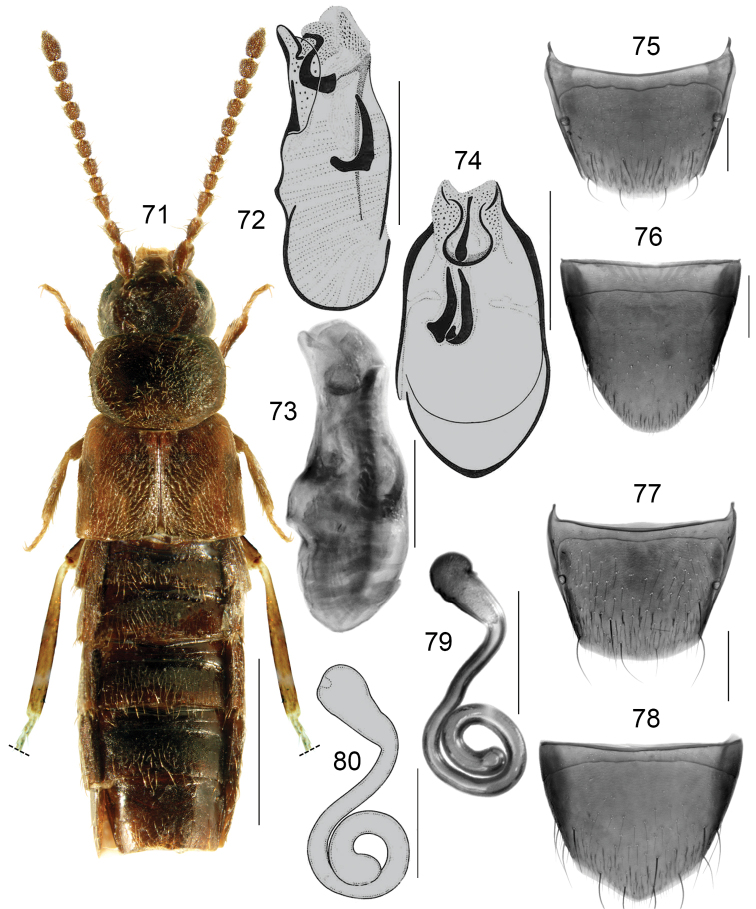
*Boreophilianeoinsecuta* Klimaszewski: **71** habitus **72, 73** median lobe of aedeagus in lateral view **74** median lobe of aedeagus in dorsal view **75** male tergite VIII **76** male sternite VIII **77** female tergite VIII **78** female sternite VIII **79, 80** spermatheca. Scale bars: 1 mm (for habitus); 0.2 mm (remaining).

###### Etymology.

Derived from prefix *neo*- added to existing specific name *insecuta*, a closely related species.

###### Diagnosis.

Body moderately broad, subparallel, forebody moderately glossy, abdomen slightly more so (Fig. 71); length 3.2–3.5 mm; head, pronotum and abdomen except for its apex dark brown, elytra reddish brown, mottled with some darker spots, appendages reddish brown; antennomeres VIII–X subquadrate; pronotum ca. as long as elytra at suture, maximum width of pronotum ca. equals to maximum width of elytra. **Male**. Tubus of median lobe of aedeagus almost straight laterally, apex narrow and rounded, bulbus broad and with two elongate and posteriorly bent sclerites (Figs 72, 73), in dorsal view median lobe elongate oval (Fig. 74); tergite VIII arcuate apically (Fig. 75); sternite VIII elongate, parabolic apically (Fig. 76). **Female**. Spermatheca: capsule club-shaped, moderately long and rounded apically, with small apical invagination; stem sinuate and coiled posteriorly (Figs 79, 80); tergite VIII arcuate apically (Fig. 77); sternite VIII produced apically and pointed medially, antecostal suture slightly sinuate laterally (Fig. 78).

###### Distribution.

Nearctic, Canada: MB, YT: USA: AK.

###### Collection data.

Habitat: tundra, under rocks. Collecting methods: forceps/aspirator. Collecting period: May to July.

###### Comments.

Lohse, in Lohse et al. (1990) reported *Boreophiliainsecuta* (Eppelsheim) in North America from AK, MB, YT, as a Holarctic species. However, these records represented a different and undescribed Nearctic species, which is here described as *B.neoinsecuta* sp. n. The two species, *B.insecuta* and *B.neoinsecuta*, have similarly shaped genitalia, which explains why they were confused. The true *B.insecuta* (Eppelsheim) was illustrated by Brundin (1954), and here (Figs 82, 83), and has a distinctly dilated apex of median lobe of aedeagus in lateral view (Fig. 82), while it is narrower in *B.neoinsecuta* (Figs 72, 73), and the two sclerites of internal sac of median lobe of aedeagus are broader and differently shaped than those of *B.neoinsecuta* (Figs 82, 83). Spermathecae of both species are very similar in shape, but female sternite VIII in *B.insecuta* is apically rounded and truncate medially, while in *B.neoinsecuta* is triangularly produced apically and pointed medially (Fig. 78).

**Figures 81–83.**
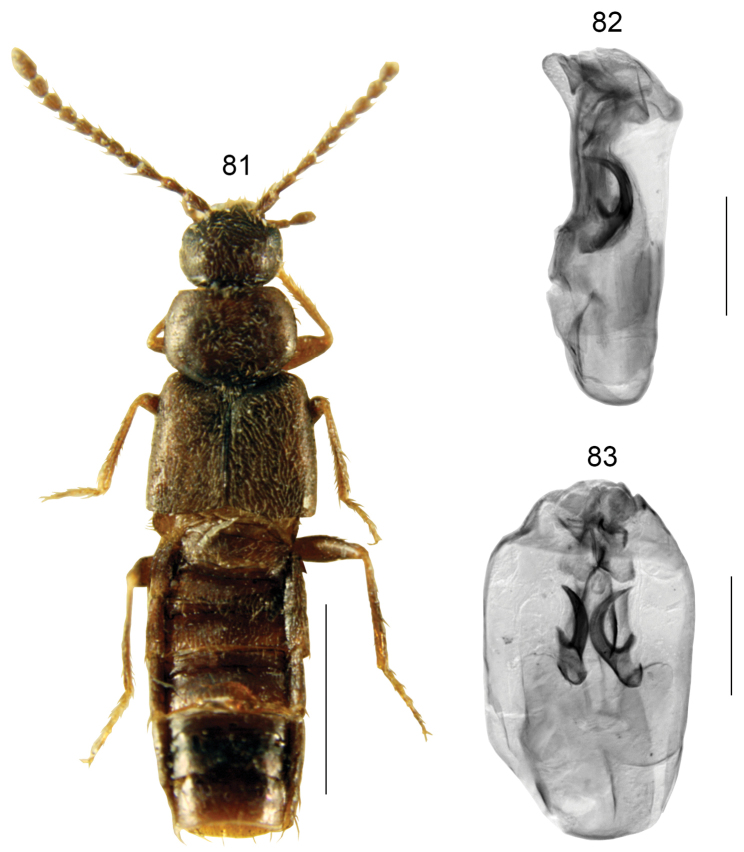
*Boreophiliainsecuta* (Eppelsheim) [specimen from Siberia, Russia]: **81** habitus **82** median lobe of aedeagus in lateral view **83** median lobe of aedeagus in dorsal view. Scale bars: 1 mm (for habitus); 0.2 mm (remaining).

###### DNA Barcode data.

Our data included two sequences of *B.neoinsecuta* paratypes, both from Alaska which grouped into BIN BOLD:ADR7545. These sequences are 0.00% distant from each other and BOLD reports they are 7.23% distant from their nearest neighbor.

##### 10. Boreophilia beringi

Taxon classificationAnimaliaColeopteraStaphylinidae

Klimaszewski & Brunke sp. n.

http://zoobank.org/2CC0CBCF-99F6-49FC-89D4-DBF5A2761DE2

Figs 84–91

###### Holotype (male).

USA, Alaska, Bering Land Bridge N. Pk., 413 m el, 65.83713N, 164.58995W, +/- 30 m snowfield, tundra, under rocks, on moss, 9.VII.2016, DS Sikes et al., UAM100418913 (UAM).

###### Paratypes.

all labelled the same except: UAM100418886 (UAM) 1 male, UAM100418887 (UAM) 1 female, UAM100418892 (UAM) 1 female, UAM100418905 (UAM) 1 male, UAM100418912 (UAM) 1 female, UAM100418905 (UAM) 1 male.

###### Etymology.

Named after Danish explorer Vitus Bering, whose name is shared with the species’ type locality, Bering Land Bridge National Park, and ‘Beringia’, the area of adjacent Russia and Alaska that were previously connected multiple times during the past 1 million years.

###### Diagnosis.

**Figures 84–91.**
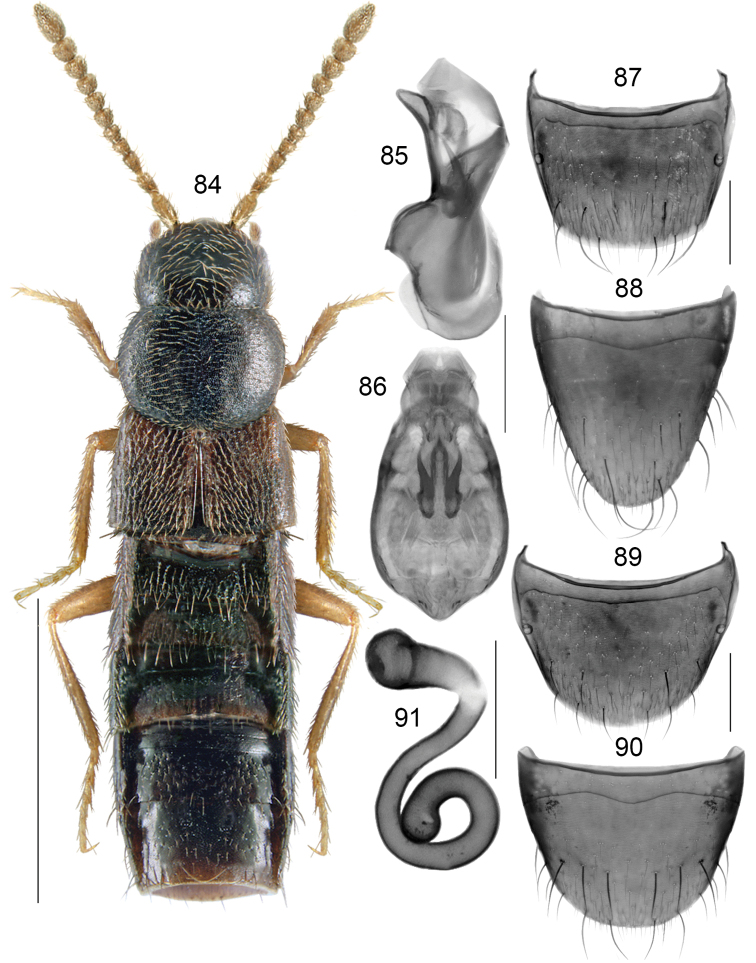
*Boreophiliaberingi* Klimaszewski and Brunke, sp. n.: **84** habitus **85** median lobe of aedeagus in lateral view **86** median lobe of aedeagus in dorsal view **87** male tergite VIII **88** male sternite VIII **89** female tergite VIII **90** female sternite VIII **91** spermatheca. Scale bars: 1 mm (for habitus); 0.2 mm (remaining).

Body narrow, subparallel, glossy, abdomen slightly more so; microsculpture of forebody strong (Fig. 84); length 2.2–2.4 mm; head, head, pronotum and abdomen dark brown, elytra with reddish brown tinge, legs paler, yellowish brown; antennomeres VIII–X subquadrate; pronotum slightly longer than elytra at suture, maximum width of pronotum ca. the same as maximum width of elytra. **Male**. Tubus of median lobe of aedeagus straight basally and produced ventrad apically in lateral view, apex triangular in shape, distinctly pointed ventrad (Fig. 85), bulbus broad in dorsal view and with two elongate sclerites, strongly narrowed at one end as illustrated (Fig. 86); tergite VIII broadly arcuate apically (Fig. 87); sternite VIII elongate, narrowly rounded apically (Fig. 88). **Female**. Spermatheca: capsule club-shaped, moderately long and rounded apically, with small apical invagination; stem sinuate and coiled posteriorly (Fig. 91); tergite VIII arcuate apically (Fig. 89); sternite VIII broadly rounded apically, antecostal suture strongly sinuate medially (Fig. 90).

###### Distribution.

Nearctic, USA: AK.

###### Collection data.

Habitat: snowfield, tundra, under rocks, on moss. Collecting methods: aspirating from moss. Collecting period: July.

###### Comments.

We here compared Palaearctic *Boreostibapiligera* (J. Sahlberg), two males from Finland (ZMUO, NHMD), with our new species from Alaska. The two species are very similar externally but may be distinguished by the different morphology of the median lobe of aedeagus. In *B.piligera*, the apical part of the tubus is more weakly deflexed ventrad and distinctly more elongate. The sclerites of the internal sac are also more elongate and quite differently shaped (less like a talon and more even in thickness along their length). Therefore we conclude that these two populations represent sibling species.

##### 11. Boreophilia subplana

Taxon classificationAnimaliaColeopteraStaphylinidae

(J Sahlberg, 1880)

Figs 92–99

Atheta subplana J Sahlberg, 1880: 90. Lohse et al. 1990: 159; Gusarov 2003: 83 [as B.angusticornis]; Smetana 2004: 396.

###### Diagnosis.

**Figures 92–99.**
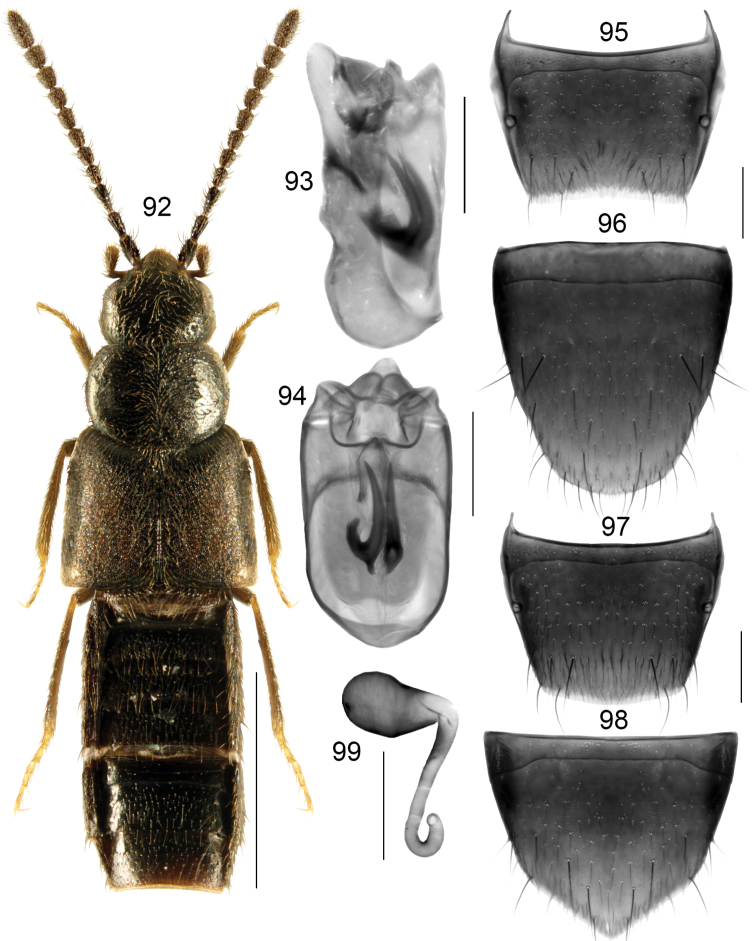
*Boreophiliasubplana* (J Sahlberg): **92** habitus **93** median lobe of aedeagus in lateral view **94** median lobe of aedeagus in dorsal view **95** male tergite VIII **96** male sternite VIII **97** female tergite VIII **98** female sternite VIII **99** spermatheca. Scale bars: 1 mm (for habitus); 0.2 mm (remaining).

Body moderately broad, strongly glossy, abdomen slightly more so (Fig. 92); length 2.8–3.5 mm; black, elytra with some reddish tinge, tarsi yellowish brown; antennomeres VIII-X slightly elongate; pronotum shorter than elytra at suture, maximum width of pronotum distinctly less than maximum width of elytra. **Male**. Tubus of median lobe of aedeagus almost straight and slightly produced ventrad in lateral view, apex broad and rounded (Fig. 93), bulbus broad and with two elongate sclerites of distinctive shape (Figs 93, 94); tergite VIII truncate or slightly concave apically (Fig. 95); sternite VIII elongate, parabolic apically (Fig. 96). **Female**. Spermatheca: capsule broadly club-shaped, moderately long and rounded apically, with small apical invagination; stem sinuate, straight medially, and looped posteriorly (Fig. 99); tergite VIII arcuate apically (Fig. 97); sternite VIII triangularly produced apically and pointed medially, antecostal suture arcuate, straight medially (Fig. 98).

###### Distribution.

Holarctic species, recorded from Spitsbergen, Fennoscandia, Russia (west and east Siberia); Canada: NT, NU; USA: AK, NH.

###### Collection data.

Habitat: *Salix* leaf litter, tundra hillside, Black spruce forest, brackish shoreline, under rocks, wrack. Collecting methods: Malaise traps, aspirating from under rocks/cobbles, sweeping low vegetation, pitfall traps. Collecting period: June to August.

###### Additional material examined.

**NEARCTIC: Canada**, NT, Muskox L., NWT, 64.45N, 108.10W, 2.VIII.1953, JG Chillcott, *Boreophiliasubplana* Sahlb. Det. GA Lohse (MHNG) 1 male; Spence Bay, NWT, 2.VII.1951, *Boreophiliasubplana* Sahlb. Det. GA Lohse (MHNG) 1 sex?

**USA**, Alaska: Anaktuvuk Pass, 647 m el., 68.14049N, 151.74091W, 19.V.2016, +/- 250 m, *Salix* leaf litter, Berlese funnel, D Sikes, K Daly, UAM100432806, UAM100432826, UAM100432833, UAM100431905, UAM100431909, UAM100413051, UAM100413054, *Salix*, sweeping, UAM100427774 (UAM) 4 males, 4 females; Anaktuvuk Pass, 665 m el., 68.14059N, 151.74266W, 20.V.2016, +/- 200 m, *Salix*, tundra hillside, pollinator pitfall, UAM100427686, UAM100427688, UAM100427690, UAM100427691, UAM100427692 (UAM) 2 males, 3 females; Tetlin NWR, Alaska Hwy, 63.36124N, 141.96761W, +/- 10 m, 13–24.VII.2015, Black spruce, Malaise, J Hagelin, UAM100391356 (UAM) 1 male; Tetlin NWR, Alaska Hwy site 2, 523 m el, 63.08456N, 141.96761W, +/- 10 m, 13–24.VII.2015, Black spruce, hanging Malaise, J Hagelin, UAM100390752 (UAM) 1 female; Kotzebue, 1 m el, 66.89151N, 162.5933W, +/- 100 m el, brackish shoreline, under rocks, wrack, 6.VII.2016, D Sikes, K Daly, UAM100420026 (UAM) 1 female; Anaktuvuk Pass, Eleanor Lk., 649 m el., 68.14742N, 151.71854W, +/- 100 m lake shore, pond, sweep, dipnet, 20.V.2016, D Sikes, K Daly, UAM100427750, UAM100427751 (UAM) 2 males.

**PALEARCTIC: Russia**, Polarnyi Ural, c. Tobols. Gyub. [ernia], F Zajzew, 5.VI.1909, A. subplana, det. Benick (MHNG) 1 male.

###### Comments.

Bernhauer (1907) described Atheta (Metaxya) angusticornis from Mount Washington, New Hampshire, USA. Gusarov (2003) transferred it to the genus *Boreophilia*, and considered it closely related to *B.subplana*, from which he differentiated it by the “shape of aedeagus, particularly a narrower apex of the median lobe in parameral view”. We have studied the median lobe of AK and NH specimens and found no differences warranting different species recognition. We therefore consider the two populations as belonging to the same species. The Mount Washington, NH, population represents a remnant, southernmost population of this species known only from higher elevations.

###### DNA Barcode data.

Our data included two sequences of *B.subplana*, both from Finland but because they are < 500 bp in length, they were not assigned to a BIN on BOLD. Our calculations indicate these sequences have an average and within-species maximum distance of 0.0% and are 6.37% distant from their nearest neighbor.

##### 12. Boreophilia caseyi

Taxon classificationAnimaliaColeopteraStaphylinidae

Lohse, 1990

Figs 100–108

Boreophilia caseyi Lohse, in Lohse et al. 1990: 155. **Holotype** (male): USA, Alaska, Umiat, 5.VIII.1950, R Madge, Boreophiliacaseyi Lohse, CNC No. 20309 (CNC) (fig. 0). **Paratypes**: USA, Alaska, Cape Thompson, 21.VII.1961, R Madge, CNC No. 20309 (CNC) 1 female; Canada, NWT, Wharton Lk., 63°52'N, 99°45'W, 18.VII.1966, JG Chillcott, CNC No. 20309 (CNC) 1 female; Canada, YT, North Fork Pass, Ogilvie Mts., 3500’, 18.VI.1962, RE Leech, CNC No. 20309 (CNC) 1 female (figs 0).
Boreophilia manitobensis Lohse 1990, in Lohse et al. 1990. **New synonymy**. Canada: MB; USA: AK. **Holotype** (male): Canada, Manitoba, Churchill, 29.VI.1937, WJ Brown, CNC No. 20311 (CNC). **Paratypes**: USA, Alaska, Umiat, 12.VII.1959, JEH Martin (CNC) 1 sex undetermined; Manitoba, Churchill, 17.VI.1952, JG Chillcott (CNC) sex undetermined.

###### Diagnosis.

**Figures 100–108.**
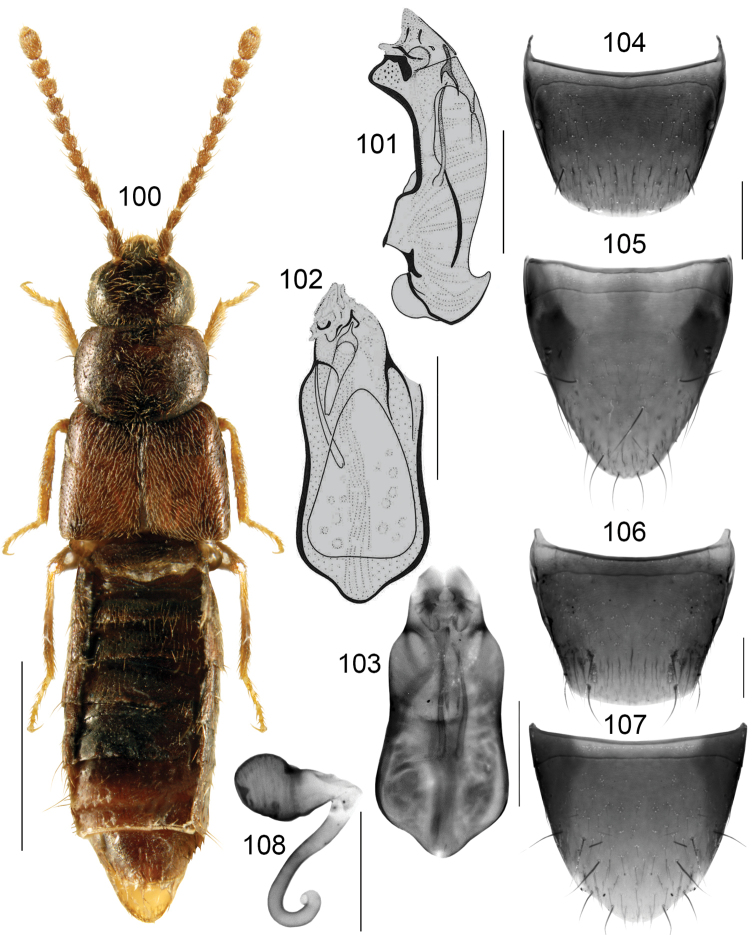
*Boreophiliacaseyi* Lohse: **100** habitus **101** median lobe of aedeagus in lateral view **102, 103** median lobe of aedeagus in dorsal view **104** male tergite VIII **105** male sternite VIII **106** female tergite VIII **107** female sternite VIII **108** spermatheca. Scale bars: 1 mm (for habitus); 0.2 mm (remaining).

Body narrow, subparallel, moderately glossy, abdomen slightly more so (Fig. 100); length 3.5–3.8 mm; head, pronotum and abdomen, except for its apex, dark brown, elytra reddish brown, legs yellowish red-brown, or body uniformly piceous with tarsi and tibiae reddish brown; antennomeres VIII-X subquadrate (females) to slightly elongate (males); pronotum as long as elytra at suture or slightly shorter, maximum width of pronotum slightly less than maximum width of elytra. **Male**. Tubus of median lobe of aedeagus straight basally and strongly projecting ventrad at apex, apex broad and angular in lateral view (Fig. 101), in dorsal view bulbus broad and angular apico-laterally, with two elongate narrow sclerites of internal sac (Figs 102, 103); tergite VIII arcuate apically (Fig. 104); sternite VIII elongate, parabolic apically (Fig. 105). **Female**. Spermatheca: capsule pitcher-shaped basally with subspherical apical part bearing small invagination, stem sinuate, narrow, looped posteriorly (Fig. 108); tergite VIII arcuate apically (Fig. 106); sternite VIII rounded apically, antecostal suture straight medially and sinuate laterally (Fig. 107).

The spermatheca of *B.caseyi* was illustrated in Lohse et al. (1990). It is slightly deformed and based on a female captured in a different locality than that of the male holotype. It may belong to *B.subplana*, a species with very similar spermatheca. The spermathecal stem of *B.caseyi* has a broad posterior loop and female sternite VIII is rounded apically (Fig. 108), and not triangularly produced and pointed medially as in *B.subplana* (Fig. 99).

###### Distribution.

Nearctic species, recorded from Canada: MB, NT, YT; USA: AK.

###### Collection data

. Habitat: arctic tundra. Collecting methods: pitfall traps. Collecting period: June and July.

###### Additional material examined.

**USA**, Alaska, Toolik Lake Field Station, 724 m el., 68.6286N, 149.59772W, +/- 6m arctic tundra, 3 pitfalls, 2.VI–30.VII.2008, D Sikes UAM100044717, UAM100044680, UAM100044997 (UAM) 2 males, 1 female.

###### Comments.

Lohse, in Lohse et al. (1990) described the new species *B.manitobensis* from MB and AK. The holotype from MB is represented by a male with a distorted median lobe of the aedeagus. We have studied the external and internal morphology of the two species, including the structures of internal sac, and found no significant differences between *B.manitobensis* and *B.caseyi*. Therefore, *B.manitobensis* is here synonymized with *B.caseyi*. The two species were published in the same paper, but *B.caseyi* has page priority and therefore was chosen as a valid species.

###### DNA Barcode data.

Four specimens of *B.caseyi* from UAM were submitted for DNA barcoding and three did not produce DNA sequences. The one which was successfully sequenced was flagged on BOLD as possibly contaminated so we excluded it from our analyses.

##### 13. Boreophilia vega

Taxon classificationAnimaliaColeopteraStaphylinidae

(Fenyes, 1920)

Figs 109–116

Atheta vega Fenyes, 1920: 198. Brundin 1954: 417, Smetana 2004: 396.
Boreophilia herschelensis Klimaszewski & Godin, in Klimaszewski et al. 2012: 232. **New synonymy**.

###### Diagnosis.

**Figures 109–116.**
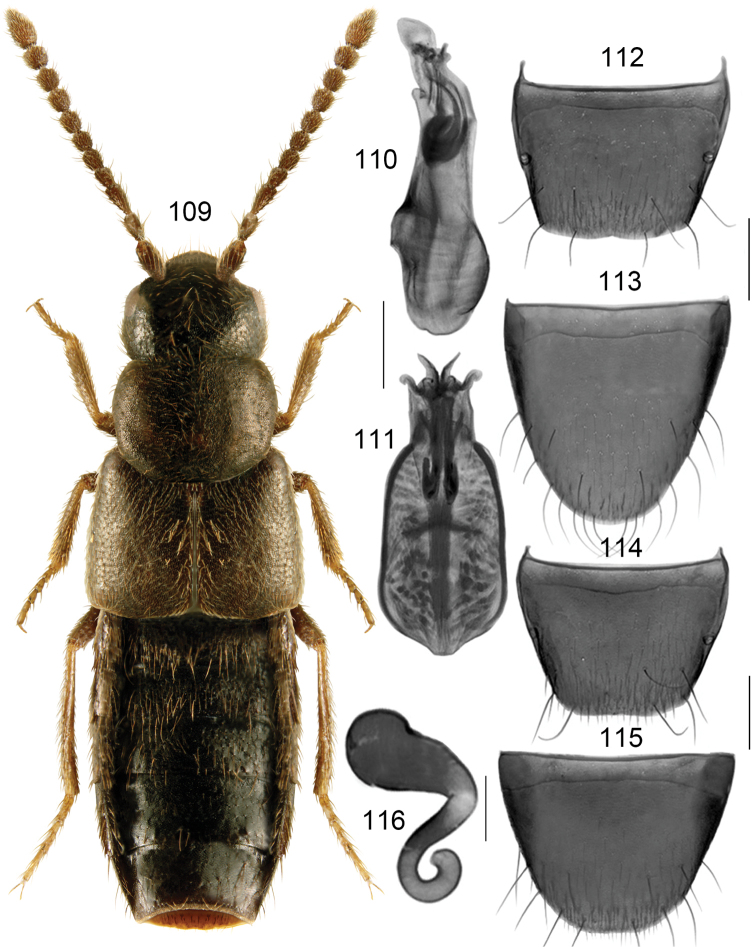
*Boreophiliavega* (Fenyes): **109** habitus **110** median lobe of aedeagus in lateral view **111** median lobe of aedeagus in dorsal view **112** male tergite VIII **113** male sternite VIII **114** female tergite VIII **115** female sternite VIII **116** spermatheca. Scale bars: 1 mm (for habitus); 0.2 mm (remaining).

Body broad, narrowly oval, moderately glossy, abdomen slightly more so (Fig. 109); length 2.8–30 mm; uniformly dark brown to almost black, tarsi paler, yellowish-brown; antennomeres VIII-X subquadrate to slightly transverse; pronotum as long as elytra at suture, maximum width of pronotum distinctly shorter than maximum width of elytra. **Male**. Tubus of median lobe of aedeagus slightly arcuate in lateral view, apex narrowly rounded, bulbus broad and with two elongate narrow sclerites (Fig. 110), in dorsal view median lobe of aedeagus elongate oval (Fig. 111); tergite VIII truncate apically (Fig. 112); sternite VIII elongate, parabolic apically (Fig. 113). **Female**. Spermatheca: capsule broadly club-shaped, moderately long and rounded apically, with small apical invagination; stem short, sinuate and looped posteriad (Fig. 116); tergite VIII truncate apically (Fig. 114); sternite VIII rounded apically and truncate medially, antecostal suture arcuate and slightly sinuate laterally (Fig. 115).

###### Distribution.

Holarctic species, known from West and East Siberia, Russian Far East, North Korea; and Canada: Herschel Island, YT. USA: not recorded.

###### Collection data.

Habitat: Yukon specimens were collected in an alluvial fan in June and July (Klimaszewski et al. 2012).

###### Comments.

*Boreophiliaherschelensis* is conspecific with *B.vega* and is here synonymized. *Boreophiliavega* has a median lobe of aedeagus similar to that of *B.neoinsecuta* (Fig. 72) and *B.insecuta* (Fig. 82), but the spermatheca of *B.vega* compared to the latter two species is differently shaped (Figs 79, 80, 116). Externally, *B.vega* differs from *B.neoinsecuta* in elytra distinctly broader that the maximum width of pronotum (Figs 71, 109). Female sternite VIII is differently shaped in the two species (Figs 78, 115).

###### DNA Barcode data.

Our data included one sequence of *B.vega* from Yukon Territory, Canada, but because this sequence was < 500 bp long it was not assigned a BIN on BOLD. We calculate that this sequence is 6.5 % distant from its nearest neighbor.

##### 14. Boreophilia davidgei

Taxon classificationAnimaliaColeopteraStaphylinidae

Klimaszewski & Godin, 2012

Figs 117–120

Boreophilia davidgei Klimaszewski & Godin, in Klimaszewski et al. 2012: 232.

###### Diagnosis.

**Figures 117–120.**
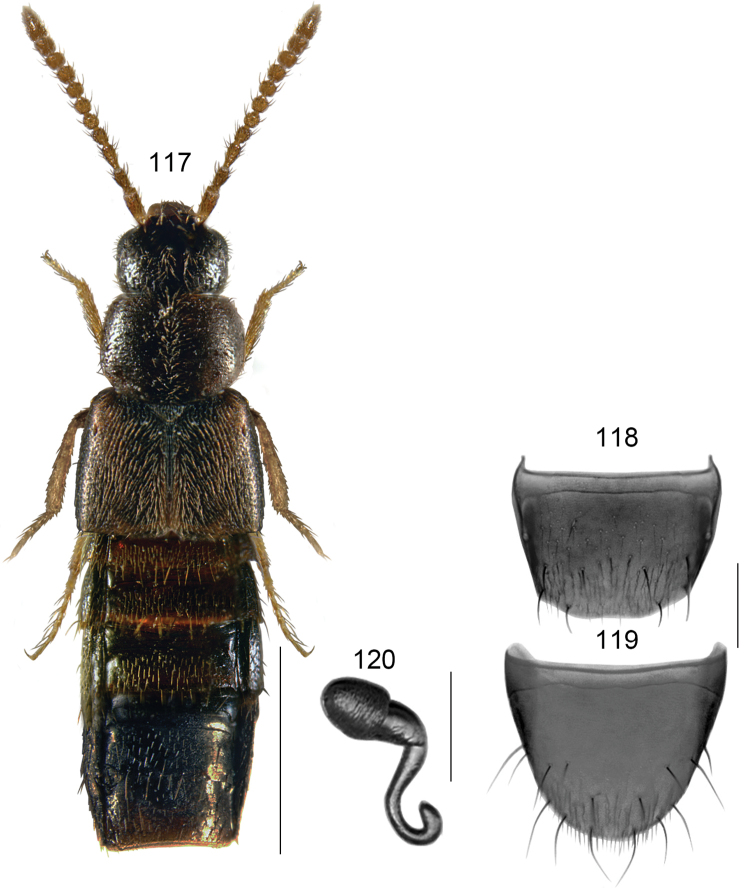
*Boreophiliadavidgei* Klimaszewski and Godin: **117** habitus **118** female tergite VIII **119** female sternite VIII **120** spermatheca. Scale bars: 1 mm (for habitus); 0.2 mm (remaining).

Body moderately broad, subparallel, moderately glossy, abdomen slightly more so (Fig. 117); length 2.8–2.9 mm; uniformly dark brown to almost black, basal sternite slightly reddish-brown, legs paler, yellowish-brown; antennomeres VIII–X subquadrate to slightly transverse; pronotum slightly shorter than elytra at suture, maximum width of pronotum distinctly less than maximum width of elytra. **Male**. Unknown. **Female**. Spermatheca: capsule ovoid apically and pitcher-shaped basally, without apical invagination; stem short, narrow, sinuate and looped posteriad (Fig. 120); tergite VIII truncate apically (Fig. 118); sternite VIII rounded apically, antecostal suture arcuate and slightly sinuate laterally (Fig. 119).

This species may be distinguished by the unique shape of spermatheca.

###### Distribution.

Nearctic species, known only from Canada, YT.

###### Collection data.

Habitat: white spruce and feathermoss forest, mixed pine and willow forest, black spruce stand, mixed aspen and white spruce forest (Klimaszewski et al. 2012). Females from Yukon were collected in May to September using pitfall traps and litter sifting in mature forest.

###### Comments.

This species is tentatively assigned to this group, because the male is unknown and morphology of median lobe of aedeagus could not be analysed.

## Supplementary Material

XML Treatment for Boreophilia

XML Treatment for Boreophilia eremita

XML Treatment for Boreophilia islandica

XML Treatment for Boreophilia fusca

XML Treatment for Boreophilia hyperborea

XML Treatment for Boreophilia nearctica

XML Treatment for Boreophilia ovalis

XML Treatment for Boreophilia nomensis

XML Treatment for Boreophilia venti

XML Treatment for Boreophilia neoinsecuta

XML Treatment for Boreophilia beringi

XML Treatment for Boreophilia subplana

XML Treatment for Boreophilia caseyi

XML Treatment for Boreophilia vega

XML Treatment for Boreophilia davidgei
